# Young cardiac telocyte-derived exosomes rejuvenate aging hearts in rats

**DOI:** 10.3389/fcell.2026.1824533

**Published:** 2026-07-09

**Authors:** Luocheng Lv, Yu Zhu, Yanjun Chen, Xin Zheng, Ruijin Huang, Hui Zhao, Ziqiang Yuan, Shanshan Feng, Xufeng Qi, Yilin Chen, Zhaofu Liao, Dongqing Cai

**Affiliations:** 1 The First Affiliated Hospital, Key Laboratory of Regenerative Medicine, Ministry of Education, Jinan University, Guangzhou, China; 2 Key Laboratory of Regenerative Medicine, Ministry of Education, Jinan University, Guangzho, China; 3 Joint Laboratory for Regenerative Medicine, Chinese University of Hong Kong-Jinan University, Guangzhou, China; 4 International Base of Collaboration for Science and Technology (JNU), Ministry of Science and Technology, Guangzhou, Guangdong, China; 5 Department of Developmental and Regenerative Biology, Jinan University, Guangzhou, China; 6 Department of Neuroanatomy, Institute of Anatomy, University of Bonn, Bonn, Germany; 7 Department of Anatomy and Molecular Embryology, Institute of Anatomy and Cell Biology, University of Freiburg, Freiburg, Germany; 8 Stem Cell and Regeneration TRP, School of Biomedical Sciences, Chinese University of Hong Kong, Hong Kong, Hong Kong SAR, China; 9 Department of Medical Oncology, Cancer Institute of New Jersey, Robert Wood Johnson of Medical School, New Brunswick, NJ, United States; 10 Dongguan Key Laboratory of Aging and Anti-Aging, Guangdong Provincial Key Laboratory of Medical Immunology and Molecular Diagnostics, Guangdong Medical University, Dongguan, China

**Keywords:** cardiac aging, cardiac telocytes, degenerative myocardiopathy, rejuvenation, young cardiac telocyte-derived exosomes

## Abstract

**Background and objective:**

Effective clinical strategies for rejuvenating aging hearts are needed to reverse and cure aging-related pathological cardiac degeneration. The present study was designed to investigate the potential rejuvenating effects of young cardiac telocyte (CT)-derived exosomes (Y-CT-exos) on cardiac aging and aging-related pathological cardiac degeneration.

**Methods:**

Y-CT-exos were prepared from young CTs. Twenty-four-month-old female Sprague‒Dawley rats were used as an aged model to evaluate the benefits of Y-CT-exos on cardiac aging and aging-related pathological cardiac degeneration. Nanoparticle tracking analysis, zeta potential measurements, Western blotting, qPCR, β-gal, ROS and PKH26 staining, multiplex immunoassays, Masson’s trichrome and immunohistochemical staining, motor function tests, wheat germ agglutinin staining and echocardiography, etc., were performed to evaluate the quality and distribution of the Y-CT-exos and their effects against cardiac aging. RNA sequencing was performed to compare the changes in the transcriptomes between aged hearts and Y-CT-exos-treated aged hearts, and differentially expressed genes (DEGs) were identified. Ingenuity pathway analysis (IPA) was used to identify related genes, their associated pathways, and the up- and downstream interaction networks underlying the rejuvenating effects of Y-CT-exos on cardiac aging and cardiac pathological improvement.

**Results:**

Y-CT-exos rejuvenated cardiac aging by ameliorating the senescence of cardiomyocytes and cardiac fibroblasts, the accumulation of DNA and ROS damage in cardiomyocytes, inflammation in the hearts and body of aged rats, promoting the proliferation of cardiomyocytes, improving aging-related decreases in cardiac function, and alleviating cardiomyocyte hypertrophy and cardiac fibrosis. In addition, the related genes, their associated pathways and the up- and downstream interaction networks underlying the therapeutic effects of reversed cellular senescence (inhibition of the p38 MAPK signaling pathway and activation of the antioxidant function of vitamin C), inflammatory aging (inhibition of the inflammasome pathway), and cardiomyocyte hypertrophy, were revealed.

**Conclusion:**

Y-CT-exos, the identified genes and their up- and downstream interaction networks, which are involved in the alleviation of cellular senescence, inflammatory aging and cardiomyocyte hypertrophy, have great potential for the development of novel cell-free therapies to rejuvenate aging hearts and ameliorate the aging-related decrease in cardiac function, cardiac hypertrophy and cardiac fibrosis.

## Introduction

Currently, cardiovascular disease (CVD) remains the leading cause of morbidity and mortality worldwide, and its prevalence increases sharply with age (Fang et al., 2025; Roth et al., 2020). Cardiac aging and aging-related deterioration in cardiac structure and function manifest as left ventricular hypertrophy, cardiac fibrosis, extracellular matrix remodeling, diastolic dysfunction, increased incidence of arrhythmia and arterial stiffness, impaired endothelial function, and reduced cardiac reserve and systolic function (Abdellatif et al., 2023; Li et al., 2025). Although current interventions targeting cardiac aging, including caloric restriction (Taffet et al., 1997; Kemi et al., 2000; Meyer et al., 2006; Riordan et al., 2008; Dai et al., 2014) and targeted pharmaceuticals such as rapamycin (Schreiber et al., 2019; Bitto et al., 2016) and senolytics (Chen MS. et al., 2022; Evangelou et al., 2023; Chang et al., 2016), have shown great potential and promise in preclinical models, an effective clinical strategy to rejuvenate aging hearts that can reverse and cure aging-related pathological degeneration is urgently needed. Cellular senescence has emerged as a pivotal contributor to the deleterious effects of cardiac aging pathophysiology and CVD (Abdellatif et al., 2025; Childs et al., 2018; Xu et al., 2018; Kumar et al., 2024; Tchkonia et al., 2013; Hall and LAJTjoca, 2024; Minamino and Komuro, 2007). In the heart, senescent cells contribute to chronic inflammation, tissue dysfunction, and reduced regenerative capacity, exacerbating cardiac dysfunction and cardiovascular conditions that are associated with aging (Hall and LAJTjoca, 2024; Minamino and Komuro, 2007). Therefore, strategies that target the rejuvenation of senescent cell phenotypes are likely to be helpful for alleviating cardiac aging.

Recent progress has demonstrated that extracellular vesicles (EVs), including exosomes that are released by all living cells into extracellular fluids (Conigliaro et al., 2017), contain many specific proteins, mRNAs, miRNAs, and long noncoding RNAs (Zomer et al., 2010; Alibhai et al., 2018) and play vital roles in the communication between the source and target cells and the regulation of aging and aging-related pathology and disease (Zhang et al., 2017). In addition, senescent cells produce small EVs containing several senescence-associated secretory phenotype (SASP) factors, which act as senescence effectors in the microenvironment (Jakhar and KJIjoms, 2019; Tanaka and Takahashi, 2021; Terlecki-Zaniewicz et al., 2018). Furthermore, EVs from young cells, such as young stem cells, can rejuvenate other cells (Kulkarni et al., 2018). EVs isolated from young human fibroblasts have rejuvenating or reparative functions that have been shown to ameliorate cellular senescence both *in vitro* and *in vivo* (Fafián-Labora et al., 2020). Plasma-derived EVs from young donors reverse aging-associated changes in hematopoietic stem cells (Sourki et al., 2023). In addition, the injection of young serum exosomes into old mice alters the expression patterns of aging-associated molecules to mimic those of young mice (Lee et al., 2018). Therefore, young EVs, including exosomes, have great potential to reverse and rejuvenate aging tissue and organs.

Recently, we reported that therapeutic transplantation of cardiac telocytes (CTs), a novel population of cardiac interstitial cells (Popescu, 2010; Popescu et al., 2010; Bani et al., 2010; Kostin, 2010; Gher et al., 2010; Rusu et al., 2012; Gherghiceanu and Popescu, 2011; Manole et al., 2011; Gherghiceanu et al., 2012; Rusu et al., 2017), in rats with acute myocardial infarction (MI) decreases the infarct size and improves myocardial function in the short term (2 weeks) and midterm (14 weeks) by promoting cardiac angiogenesis and reconstruction of the CT network and reducing cardiac fibrosis and cardiac degenerative myocardiopathy (Zhao et al., 2013; Zhao et al., 2014). More recently, we reported that young CT-exosomes (CT-exos) inhibit the apoptosis of cardiac microvascular endothelial cells through exosomal miRNA-21-5p-targeted *Cdip1* silencing to improve angiogenesis following MI, which promotes regeneration, alleviates degenerative myocardiopathy and improves cardiac function (Liao et al., 2021). These findings suggest that CT-exos have unique characteristics, as CTs exert therapeutic effects in terms of regenerating MI tissues, improving cardiac function and ameliorating degenerative myocardiopathy. Accordingly, we hypothesized that Y-CT-exos might have the potential to rejuvenate aging hearts by ameliorating cellular senescence and senescence-associated deleterious changes.

In the present study, we report for the first time that intramyocardial injection of Y-CT-exos into aged hearts exerts rejuvenating effects by ameliorating cardiomyocytes and cardiac fibroblasts senescence, cardiomyocyte DNA and ROS damage and aged heart and body inflammaging, improving proliferation of cardiomyocytes, age-related increasing of cardiomyocyte hypertrophy and endocardium fibrosis, and age-related decrease of cardiac function. In addition, four pathways, along with their associated genes and interaction networks, underlying the rejuvenating effects of Y-CT-exos in terms of alleviating cellular senescence, inflammation and cardiomyocyte hypertrophy were elucidated.

## Materials and methods

### Animals

Three-month-old female Sprague‒Dawley (SD) rats (200–250 g) and twenty-four-month-old female SD rats (300–500 g) were utilized in the present study. The rats were housed for 2 weeks to allow them to adapt before experimentation. They were provided with food and water *ad libitum*. Animal care and surgical and handling procedures were performed according to regulations established by The Ministry of Science and Technology of the People’s Republic of China ([2006] 398) and approved by the Jinan University Animal Care Committee (Ethics Approval Number: GZJLAWE-20190916–13).

### Isolation and phenotypic confirmation of CTs

CTs were isolated from young (3-month-old) female SD rats using our well-established method, as described in our previously published papers (Zhao et al., 2013; Zhao et al., 2014; Liao et al., 2021). With this method, more than 93% of the isolated cells were c-Kit^+^ and CD34^+^ (Zhao et al., 2013; Zhao et al., 2014; Liao et al., 2021). The phenotype of the CTs was confirmed as described in detail in our previous publication (Liao et al., 2021). The isolated CTs were cultured in DMEM supplemented with 20% fetal calf serum at 37 °C with 5% CO_2_ in a 95% air incubator.

### Purification of Y-CT-exos

Y-CT-exos were prepared from young**-**CTs. CTs were grown in 100-mm dishes at 37 °C with 5% CO_2_ and 95% air in DMEM supplemented with 1% penicillin, streptomycin and 20% FBS until they reached 80%–90% confluence, after which the culture medium was removed. After three washes with PBS (pH = 7.4), DMEM (8 mL, containing 20% exosome-free serum) was added, and the cells were incubated at 37 °C with 5% CO_2_ and 95% air for 48 h. Ten milliliters of conditioned medium was harvested from different dishes and centrifuged at 3000 *g* for 15 min to pellet the cells before the samples were filtered through a 0.22-µm filter to remove cell debris. Y-CT-exos were extracted from the collected medium with ExoQuick-TC Exosome Precipitation Solution (cat. No. EXOTC50A-1; SBI) according to the manufacturer’s instructions. Briefly, 2 mL of ExoQuick-TC Exosome Precipitation Solution was mixed with the collected medium, incubated at 4 °C overnight, and then centrifuged at 1,500 *g* for 30 min. After the supernatant was removed, the pellet was centrifuged again at 1,500 × g for 5 min to remove the residual media, after which the Y-CT-exos were resuspended in PBS (pH = 7.4).

### Transmission electron microscopy (TEM) analysis of Y-CT-exos

Three microliters of the prepared Y-CT-exos were placed on Formvar carbon-coated 200-mesh copper electron microscopy grids, incubated at room temperature for 5 min, and then subjected to standard uranyl acetate staining. The grids were washed with PBS three times and semidried at room temperature before observation. The morphology and diameter of the Y-CT-exos were analyzed using TEM (Philips Tecnai 10 TEM).

### Nanoparticle tracking analysis (NTA) of Y-CT-exos

The absolute size distributions of the Y-CT-exos were analyzed using a NanoSight NS300 instrument (Malvern, UK). The light scattered by the Y-CT-exos upon laser illumination was captured by a camera, and video files of the Brownian motion of the Y-CT-exos were created. NTA software was used to track and analyze individual particles ranging in size from 10 to 1,000 nm. Three recordings were performed for each sample.

### Zeta potential measurements of Y-CT-exos

The surface zeta potential of the Y-CT-exos was analyzed at 25 °C using a Zetasizer Nano ZSE (Malvern Instruments Ltd., UK). The zeta potentials of Y-CT-exos were measured with the following settings: a sensitivity of 85, a shutter value of 70, and a frame rate of 30 frames per second. ZetaView software was used to collect and analyze the data.

### Western blot analysis

Y-CT-exos lysates were prepared in radioimmunoprecipitation assay (RIPA) buffer (cat. No. P0013C; Beyotime) containing a protease inhibitor cocktail (cat. No. W2200s; CWBIO). The total protein concentration was analyzed using a BCA protein assay kit (cat. No. P0010; Beyotime). The extracted Y-CT-exos proteins were denatured in loading buffer (5 × SDS‒PAGE loading buffer, cat. No. 20315ES05; Yeasen Biotechnology) for 10 min at 95 °C, electrophoresed on a 12% SDS‒PAGE gel, and transferred to a PVDF membrane (cat. No. 162–0177; Bio-Rad). After being blocked with 5% nonfat milk in TBST buffer, the PVDF membranes were incubated separately with anti-CD63 (1:1,000; cat. No. Sc-5275; Santa Cruz), anti-CD81 (1:1,000; cat. No. HY-P80608; MedChemExpress), anti-ALIX1 (1:1,000; cat. No. HY-P80011; MedChemExpress), anti-CALNEXIN (1:1,000; cat. No. HY-P80041; MedChemExpress) antibodies, and anti-GAPDH (1:5,000; cat. No. YA418; MedChemExpress) antibodies overnight, after which the membranes were washed with TBST and incubated with horseradish peroxidase (HRP)-conjugated secondary antibodies. The protein bands were visualized and imaged using an enhanced chemiluminescence system and a chemiluminescence reader (GeneGnome HR; Synoptics).

### Transplantation of Y-CT-exos into aged rats

Twenty-four-month-old female SD rats were anesthetized via intraperitoneal injection of 20% urethane (0.5 ml/100 g) and placed in the supine position. After the neck and chest areas were shaved and disinfected, a midline cervical incision was made to expose the trachea. Tracheal intubation was performed, and mechanical ventilation was initiated using a small-animal ventilator (tidal volume: 1.5–2.0 ml; respiratory rate: 80 breaths/min). The thoracic cavity was opened *via* an incision between the 4th and 5th intercostal spaces on the left side to expose the heart, after which a pericardial incision was made. In the experimental group, a microsyringe was used to perform intramyocardial multipoint injections at the following locations: two sites in the anterior left ventricular wall and one site each in the left, right, and apical border zones of the anterior wall. A total volume of 10 μL (containing 60 μg of Y-CT-exos) was administered per site, resulting in a cumulative dose of 300 μg. The control group received injections of an equal volume of phosphate-buffered saline (PBS; pH = 7.4) at identical anatomical sites. After the procedure, the thoracic wall was closed layer by layer, and the surgical area was disinfected. The tracheal tube was removed once spontaneous breathing resumed, and the skin incision was sutured. Two weeks after surgery, the experimental group received a tail vein injection of 300 μg of Y-CT-exos (300 μL, 1 μg/μl), whereas the control group received an equal volume of PBS (pH = 7.4). All the animals were maintained for an additional 2 weeks, resulting in a total intervention period of 4 weeks, after which cardiac function assessments and tissue collection were performed.

### Senescence-associated beta-galactosid

#### ase (SA-β-gal) staining

Cardiac cryosections were rinsed once with 1× PBS (pH = 7.4), followed by fixation in fixative solution at room temperature for 7 min. After three washes with 1× PBS (pH = 7.4), SA-β-gal staining solution (cat. No. C0602; Beyotime Biotechnology) was added, and the sections were incubated overnight at 37 °C in a humidified chamber. After three washes with 1× PBS (pH = 7.4), a rabbit anti-cardiac cTnT (marker of cardiomyocytes; Proteintech, 15513-1-AP) antibody and a mouse anti-vimentin (marker of fibroblasts; Santa Cruz Biotechnology, sc-6260) antibody were added, and the samples were incubated overnight at 4 °C and then washed with 1× PBS (pH = 7.4) three times. FITC–conjugated goat anti-rabbit IgG (green; Proteintech, SA00003-2) and Cy3-conjugated goat anti-mouse IgG (red; Proteintech, SA00009-1) were subsequently added for 1 h of incubation. Finally, the nuclei were counterstained with DAPI. All the procedures were conducted in the dark. Following staining, the sections were washed three times with 1× PBS, mounted with 70% glycerol, and imaged under a ×20 objective lens. The numbers of β-gal-positive (blue) cardiomyocytes (green) and cardiac fibroblasts (red) in each heart section were quantified.

#### Distribution of transplanted Y-CT-exos

To investigate *in vivo* distribution and engulfment, Y-CT-exos were labeled with PKH26 (cat. No. HY-P80011; MedChemExpress). The staining reaction was terminated by treatment with 3% BSA for 1 min, and the labeled exosomes (PKH26-Y-CT-exos) were washed three times with PBS using Vivaspin 2 centrifugal concentrators (300 kDa MWCO; cat. No. VS0251, Sartorius) to remove the unbound dye. Additionally, a Mock-labeled control (PBS incubated with the same concentration of PKH26 dye and subjected to the same purification process as the Y-CT-exos) and unlabeled Y-CT-exos were used as controls. PKH26-Y-CT-exos were transplanted into rats by intramyocardial injection or tail vein injection. The heart, forelimb muscle, hind limb muscle, liver, spleen, kidney, lung and brain tissues from the treated rats were dissected at 1 h, 6 h, and 24 h after injection and then cryosectioned for subsequent observation by confocal microscopy (Imager Z2; Zeiss, Germany). The *in vivo* engulfment of Y-CT-exos at 1 h, 6 h and 24 h after intramyocardial injection of PKH26-Y-CT-exos (red) was analyzed in myocardial cryosections of individual cardiac cells using immunofluorescence staining (green) (anti-α-cardiac actin for cardiomyocytes, anti-vimentin for cardiac fibroblasts, anti-vWF for endothelial cells and anti-α-SMA for smooth muscle cells), after which the cells were observed and imaged by fluorescence microscopy (Imager Z2; Zeiss, Germany).

#### Senescence induction in H9C2 cardiomyocytes and exosome treatment

Doxorubicin (Dox; HY-15142; MedChemExpress) was used to induce senescence in H9C2 cardiomyocytes. Cells (4 × 10^4^) were cultured in DMEM supplemented with 10% fetal calf serum at 37 °C with 5% CO_2_ in a 95% air incubator for 24 h and treated with 0.2 μM Dox for 24 h to induce cellular senescence. After the medium was changed to remove the Dox, Y-CT-exos (100 μg/mL) or O-CTs-exos (100 μg/mL, derived from 24-month-old rats) were added for 96 h of treatment (the medium was replaced with fresh medium supplemented with the same amount of Y-CT-exos and O-CTs-exos after 48 h). SA-β-Gal staining was performed to evaluate the antisenescence effects of Y-CT-exos and O-CTs-exos.

#### Measurement of intracellular reactive oxygen species (ROS) levels

Intracellular ROS levels were measured by flow cytometry using the dihydroethidium (DHE; HY-D0079, MedChemExpress) probe. H9C2 cardiomyocytes (8 × 10^4^) were cultured in DMEM supplemented with 10% fetal calf serum at 37 °C with 5% CO_2_ in a 95% air incubator for 24 h and treated with 0.2 μM Dox for 24 h to induce cellular senescence. After the medium was changed to remove the Dox, Y-CT-exos (100 μg/mL) or O-CT-exos (100 μg/mL) were added for 24 h of treatment (the medium was replaced with fresh medium containing the same amount of Y-CT-exos and O-CT-exos after 48 h; the resulting group was named the Dox-Y-CT-exos group). The blank group and Dox-treated group (Dox) were used as controls. The treated cells were then stained with a DHE probe (10 μM) in serum-free DMEM for 30 min. After the unincorporated extracellular DHE probe was removed, the DHE fluorescence intensity (red) was measured using a CytoFLEX flow cytometer (Beckman Coulter). Fluorescence signals were acquired through the PE channel, with a minimum of 10,000 cellular events recorded for each group. The data were analyzed using FlowJo software (BD). The intracellular ROS levels were quantified as the mean fluorescence intensity (MFI) in the PE channel.

#### Isolation of total RNA and real-time quantitative PCR

Total RNA was extracted using TRIzol reagent (cat. No. 15596018; Invitrogen) according to the manufacturer’s protocol. To analyze the expression of cyclin-dependent kinase inhibitor 1A (p21), cyclin-dependent kinase inhibitor 2A (p16), tumor protein p53 (p53), cyclin-dependent kinase inhibitor 1B (p27), lamin B1, mechanistic target of rapamycin (mTOR), forkhead box O3 (FOXO3), silent information regulator 1 (Sirt1), interleukin-18 (IL-18), interleukin-6 (IL-6), tumor necrosis factor-alpha (TNF-α), and interleukin-1 beta (IL-1β), the extracted RNA (1 μg) was reverse transcribed into first-strand cDNA using Hifair® III 1st Strand cDNA Synthesis SuperMix for qPCR (cat. no. 118141ES; Yeasen Biotechnology) according to the manufacturer’s instructions, after which the gene expression levels were analyzed using SYBR Green-based real-time PCR. The reaction mixture was composed of 10 µL of SYBR Green PCR Master Mix (cat. No. B21202; Biotool), 1 µL of each primer, 8 µL of PCR-grade water and 1 µL of the cDNA template. The primer sequences are listed in Supplementary Table S1. Amplification reactions were performed with a Mini-Opticon System using the following conditions: 95 °C for 5 min, followed by 40 cycles of denaturation at 95 °C for 15 s and primer annealing and extension at 62 °C for 30 s. All cDNA samples were amplified in triplicate and normalized to Gapdh expression on the same plate. Three replicates of each sample were examined.

#### Multiplex immunoassays of serum inflammatory cytokines

Serum concentrations of inflammatory cytokines were quantified using a multiplex bead-based immunoassay. Briefly, venous blood samples were collected from the jugular vein of the rats and allowed to clot at room temperature for 30 min. The samples were then centrifuged at 2,000 × g for 15 min at 4 °C. The serum was aliquoted and stored at −80 °C until analysis.

The levels of multiple inflammatory cytokines—including tumor necrosis factor-alpha (TNF-α), interleukin-1 beta (IL-1β), interleukin-6 (IL-6), and interleukin-18 (IL-18) regulated upon activation normal T-cell expressed and presumably secreted (Rantes), granulocyte-macrophage colony-stimulating factor (GM-CSF), interleukin-10 (IL-10), and interleukin-13 (IL-13) were simultaneously measured using a commercially available multiplex immunoassay kit (cat. No. RECYTMAG-65K; Sigma) according to the manufacturer’s instructions. All the samples were assayed in duplicate.

#### Immunohistochemistry

For DNA damage analysis, cardiac cryosections were fixed in 4% paraformaldehyde (15 min), permeabilized with 0.5% Triton X-100 (20 min), and blocked with blocking solution (1 h). The sections were incubated overnight at 4 °C with the following primary antibodies: rabbit anti-γH2A.X (1:400; cat. No. ab11174; Abcam) and mouse anti-α-sarcomeric actinin (α-SA; 1:200; cat. No. ab9465; Abcam). After being washed, the slides were incubated with Alexa Fluor 555-conjugated donkey anti-rabbit IgG (1:500; cat. No. A-31572; Thermo Fisher Scientific) and Alexa Fluor 488-conjugated donkey anti-mouse IgG (1:500; cat. No. A-21202; Thermo Fisher Scientific) for 1 h at room temperature in the dark. The nuclei were counterstained with DAPI (Cat. No. C1002; Beyotime Biotechnology). Images were acquired using a Leica microscope (×20 objective) with sequential scanning. Quantification was performed by calculating the percentage of γH2A.X-positive cells (γH2A.X^+^/DAPI^+^) within α-SA-positive myocardial tissue. All the analyses were performed in a double-blind manner.

For proliferation analysis, cardiac cryosections were fixed in 4% paraformaldehyde (15 min), permeabilized with 0.3% Triton X-100 (20 min), and blocked with blocking solution (1 h). The sections were incubated overnight at 4 °C with the following primary antibodies: rabbit anti-cTnI (1:200; cat. No. ab19615; Abcam) and mouse anti-Ki67 (1:100; cat. No. ab8191; Abcam). After being washed, the slides were incubated with Alexa Fluor 488-conjugated goat anti-rabbit IgG (1:500; cat. No. A-11008; Thermo Fisher Scientific) and Alexa Fluor 555-conjugated goat anti-mouse IgG (1:500; cat. No. A-21424; Thermo Fisher Scientific) for 1 h at room temperature in the dark. The nuclei were counterstained with DAPI (cat. No. C1002; Beyotime Biotechnology). Images were acquired using a Leica microscope (×20 objective) with sequential scanning. Cardiomyocyte proliferation was quantified as the percentage of cTnI-positive cells with Ki67-positive nuclei. All the analyses were performed in a double-blind manner by two independent investigators.

#### Echocardiography

Transthoracic echocardiography was performed on the experimental rats using a Vevo 2100 echocardiogram (VisualSonics, Canada). The rats were anesthetized via inhaled isoflurane (induction: 4%, maintenance: 1.5%–2.0% in 100% O_2_ at 1 L/min) with a nose cone. A stable heart rate of >300 b.p.m. was maintained. The body temperature of the rats was maintained at 37 °C and 37.5 °C using a heating pad with a rectal probe. The rats were placed in the left lateral decubitus position. A 13-MHz linear transducer was placed on the shaved left chest wall and oriented to obtain standard parasternal long-axis views, followed by parasternal short-axis and views at the 19 midpapillary muscle level. Mmode tracings were recorded from the shortaxis view for measurement of the systolic and diastolic dimensions. Apical fourchamber views were obtained for Doppler measurements of mitral valve E and A waves (Zhao et al., 2013; Zhao et al., 2014; Liao et al., 2021; Chen H. et al., 2022). All procedures were performed by a single experienced operator to minimize variability.

#### Motor function test

Forelimb strength: Rats from each group were suspended on a pull-strength apparatus. Each rat grasped the pull bar with its forelimbs, and the tail was then elevated to a horizontal position to achieve full suspension. The maximal reading on the force gauge was recorded. Each rat underwent five trials at 3-min intervals, and the mean of the five measurements was calculated for statistical analysis. Endurance: Rats from each group were positioned above the grip bar of a hanging endurance tester. Each animal was allowed to grasp the bar with its forelimbs and remain suspended. The latency to fall was recorded. Five trials per rat were conducted with 3-min intervals between trials, and the mean latency across trials was used for statistical analysis. Maximum walking distance: Rats were placed on an eight-lane treadmill. After a 5-min warm-up period at 5 m/min, the speed was increased by 3 m/min every 3 min to a maximum of 29 m/min. The rats continued running at 29 m/min until exhaustion, which was defined as the failure to resume running despite electrical stimulation. The total distance ran for each rat was recorded (Justice et al., 2014).

#### Wheat germ agglutinin staining

The cardiomyocyte area was evaluated by performing wheat germ agglutinin (WGA) staining. WGA was used to label intercellular substances and the cell membrane, and cardiac troponin I (cTnI) was used to specifically mark cardiomyocytes. Hydrated sections were pretreated with Tris-EDTA buffer for antigen retrieval. Afterward, the sections were incubated with WGA (cat. No. W11261; Invitrogen) for 10 min and then washed with PBS (pH = 7.4). The sections were blocked with 1% BSA at room temperature for 1 h and then incubated with an anti-cTnI antibody (1:100; cat. No. 21652-1-AP; Proteintech) at 4 °C overnight. After being washed, the sections were incubated with an Alexa Fluor 555-conjugated donkey anti-rabbit antibody (1:500) for 1 h and then with Hoechst for 15 min. The sections were sealed with antifade mounting medium and observed under a microscope. The cross-sectional areas of the cardiomyocytes were measured using Image-Pro Analyzer 6.0 software.

#### Masson’s trichrome staining and analysis of cardiac fibrosis

The frozen-embedded sections were subjected to Masson’s trichrome staining to evaluate cardiac fibrosis according to standard protocols. Briefly, after iron hematoxylin staining (7 min), the sections were stained with Ponceau acid fuchsin (5 min) and rinsed with distilled water. After differentiation using a phosphomolybdic acid solution (5 min), the sections were sequentially stained with aniline blue (5 min) and 1% acetic acid (1 min). Afterward, the sections were dehydrated, cleared in xylene, and then mounted with resin. The area of fibrosis in the endocardium, which is the collagen area (blue), was compared among the groups using Image-Pro Analyzer 6.0 software.

#### RNA sequencing (RNA-seq) and transcriptome analysis

RNA sequencing was performed by Oebiotech Company (Shanghai, China). Total RNA was extracted from cardiac tissue using TRIzol reagent, treated with DNase I, and subjected to quality control (RIN ≥7.5). Libraries were prepared from 1 µg of RNA per sample using the NEBNext Ultra II Directional RNA Library Prep Kit, followed by paired-end sequencing (2 × 150 bp) on an Illumina NovaSeq 6,000 platform. Raw reads were quality-trimmed with Trimmomatic and aligned to the rat genome (Rnor_6.0) using HISAT2. Gene counts were generated with featureCounts. Differential expression analysis was performed with DESeq2, and DEGs were defined as genes whose |log_2_FC| was >1 and FDR was <0.05. Enrichment analysis of GO terms and KEGG pathways was conducted using clusterProfiler (FDR <0.05).

#### Ingenuity pathway analysis (IPA)

Differentially expressed genes (DEGs) were identified by comparing the transcriptomes of Y-CT-exo-treated aged hearts and PBS-treated control aged hearts at 4 weeks after Y-CT-exos treatment. The DEGs were subjected to core analysis using Ingenuity IPA. To identify significantly activated or suppressed canonical pathways, the threshold for the -log (p value) was set to >1.3 (default parameter, indicating significantly enriched pathways), and the absolute value of the z score was set to >2 (where a z score >2 denotes significant activation and a z score < −2 denotes significant suppression of a pathway).

### Statistical analysis

One-way ANOVA with the least significant difference (LSD) test was performed using SPSS version 17 software to determine the P values for the data from the repeated experiments. All values are presented as the means ± standard deviations (S.Devs). *P* < 0.05 indicated a statistically significant difference.

## Results

### Treatment with Y-CT-exos decreases the number of senescent cardiomyocytes and cardiac fibroblasts in aged hearts

**FIGURE 1:**
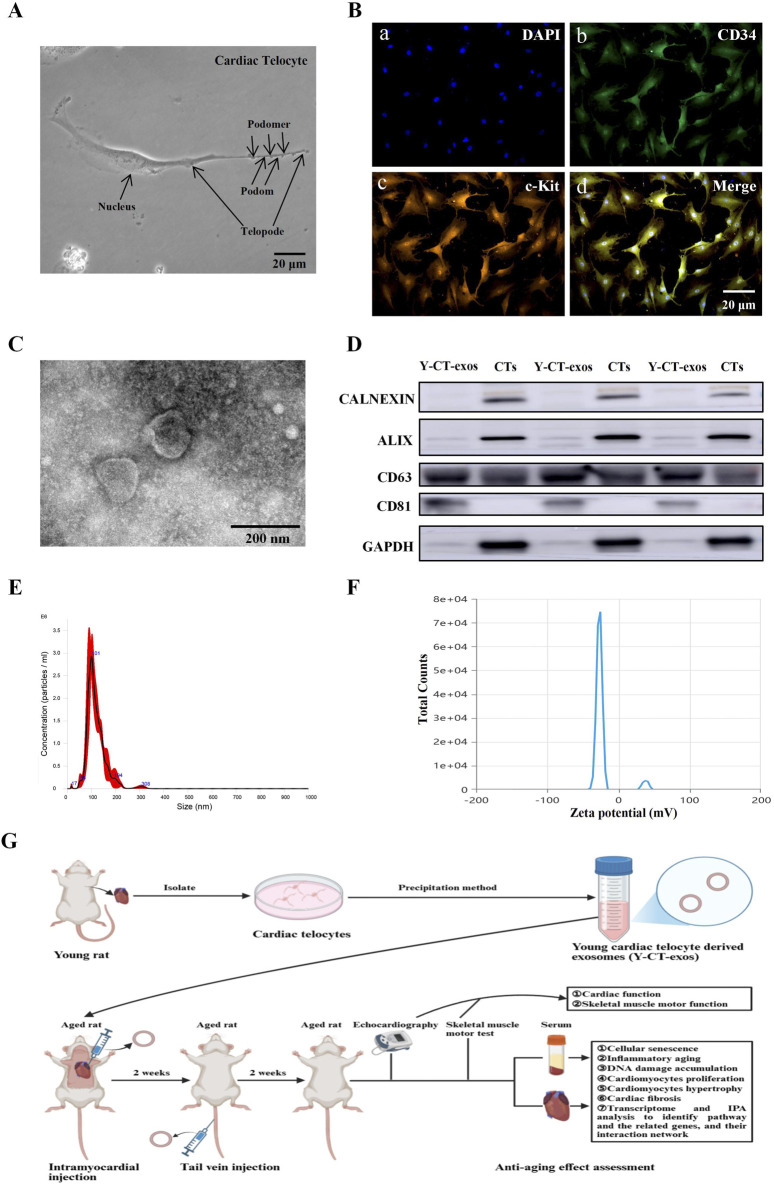
Preparation and characterization of Y-CT-exos and study design. **(A)** The isolated CTs show a unique morphology characterized by long telopodes composed of podoms and podomers. **(B)** The isolated CTs expressed the generally accepted markers c-Kit and CD34. **(C,D)** The isolated Y-CT-exos presented a unique exosomal morphology and were positive for the exosome markers CD63, CD81 and ALIX and negative for CALNEXIN (a negative exosome marker), while CT whole-cell lysate-positive controls were positive for calnexin. Y-CT-exos: Y-CT-exos lysate. CTs: CT whole-cell lysates (positive control). **(E)** NTA revealed that the diameter of the isolated Y-CT-exos was approximately 100–200 nm. **(F)** Analysis of the zeta potential revealed that the isolated Y-CT-exos were negatively charged. **(C–F)** Y-CT-exos exhibited fit the well-established characteristics of exosomes. **(G)** Graphical illustration of the study design to evaluate the effects of Y-CT-exos against cardiac aging *in vitro* and *in vivo*. Image **(G)** was created with BioRender (https://BioRender.com).

We first conducted a pilot study to compare the antisenescence effects of Y-CT-exos and old cardiac telocyte (CT)-derived exosomes (O-CT-exos) using an *in vitro* doxorubicin (Dox)-induced H9C2 cardiomyocyte senescence model combined with β-gal staining. The density of β-gal-positive cardiomyocytes in the Dox-treated group (Dox) was significantly greater than that in the blank control group, which confirmed the successful induction of cellular senescence. In addition, compared with that in the Dox-treated senescent control group, the density of β-gal-positive cardiomyocytes in the Y-CT-exos-treated group was significantly lower; however, the density of β-gal-positive cardiomyocytes in the O-CT-exos-treated group was similar to that in the Dox-treated senescent control group (Supplementary Figure S1), which suggests that Y-CT-exos treatment alleviates cardiomyocyte senescence but that O-CT-exos treatment does not. Therefore, the present study was designed to investigate the potential rejuvenating effect of Y-CT-exos on cardiac aging and aging-related pathological cardiac degeneration. Accordingly, young CTs were isolated from the hearts of young rat using our established protocol (Zhao et al., 2013; Zhao et al., 2014; Liao et al., 2021). These CTs displayed a characteristic telocyte morphology with long telopods composed of podoms and podomers (Figure 1A) (Popescu, 2010; Popescu et al., 2010; Bani et al., 2010; Kostin, 2010; Gher et al., 2010; Rusu et al., 2012; Gherghiceanu and Popescu, 2011; Manole et al., 2011; Gherghiceanu et al., 2012; Rusu et al., 2017; Zhao et al., 2013; Zhao et al., 2014; Liao et al., 2021) and expressed the generally accepted markers c-Kit and CD34 (Figure 1B) (Zhao et al., 2013; Zhao et al., 2014; Liao et al., 2021). Using these young CTs, we generated Y-CT-exos using the established method (Liao et al., 2021) described in the Methods section. The prepared Y-CT-exos presented a unique exosomal morphology (Figure 1C), and WB analysis revealed that the Y-CT-exos positively expressed the exosome markers CD63, CD81 and Alix but not calnexin (a negative exosome marker) (Figure 1D). NTA revealed that the diameter of the isolated Y-CT-exos ranged from approximately 100–200 nm (Figure 1E), and zeta potential measurements revealed that the isolated Y-CT-exos were negatively charged (Figure 1F); both of these features are consistent with the well-established diameter and surface charge of exosomes (Khan et al., 2015; Huang et al., 2019; Gao et al., 2020; Wang et al., 2021). To observe the *in vivo* distribution and engulfment of Y-CT-exos after intramyocardial or tail vein injection, Y-CT-exos were stained with a PKH26 probe before administration. The fluorescence signal (red) was detected in the cryosections of individual organs, namely, the heart, liver, spleen and lung, but not in the fore limb muscle, hind limb muscle, kidney or brain at 1 h, 6 h and 24 h after intramyocardial injection (Supplementary Figure S2). However, after tail vein injection, a fluorescence signal was observed in the liver, spleen and lung but not in the heart, forelimb muscle, hind limb muscle, kidney or brain at 1 h, 6 h or 24 h after tail vein injection (Supplementary Figure S3). In addition, *in vivo* engulfment of Y-CT-exos after intramyocardial injection of PKH26-Y-CT-exos (red) was analyzed in individual cardiac cells using immunofluorescence staining (green) (anti-α-cardiac actin for cardiomyocytes, anti-vimentin for cardiac fibroblasts, anti-vWF for endothelial cells and anti-α-SMA for smooth muscle cells). Red fluorescence signals of Y-CT-exos were detected in cardiomyocytes, cardiac fibroblasts and endothelial cells but not in smooth muscle cells (Supplementary Figures S4-7), revealing that the Y-CT-exos transplanted *via* intramyocardial injection could be engulfed at least by cardiomyocytes, cardiac fibroblasts and endothelial cells *in vivo*. Accordingly, the antiaging effects of Y-CT-exos were investigated in aged hearts both *in vitro* and *in vivo*, as illustrated in Figure 1G.

**FIGURE 2:**
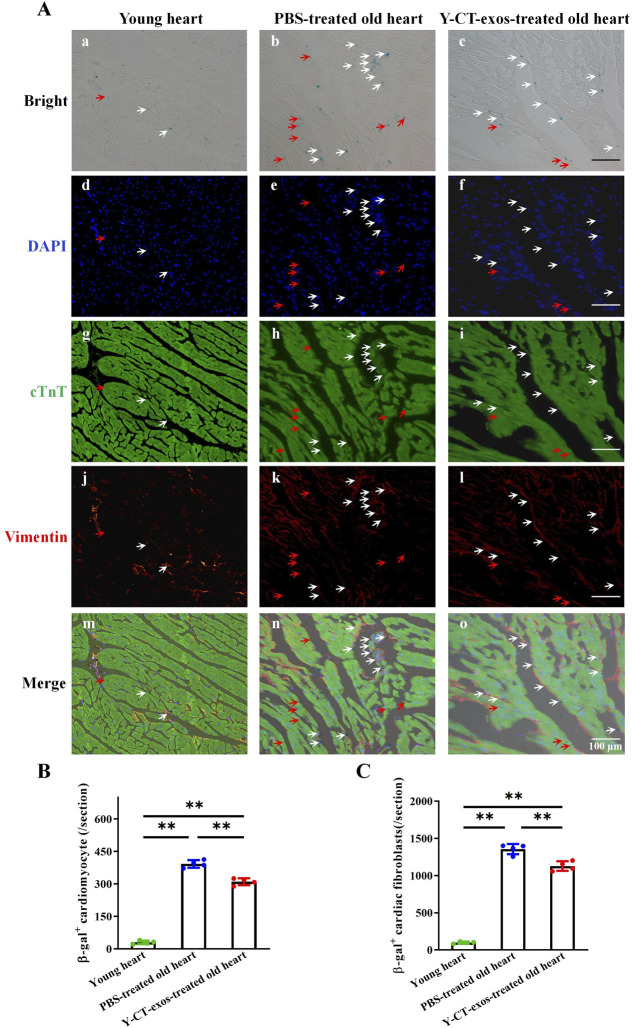
Y-CT-exos treatment decreased the number of senescent cardiomyocytes and cardiac fibroblasts in aged hearts. Representative images of β-gal-stained young hearts **(A,a)**, PBS-treated aged hearts **(A–b)** and Y-CT-exos-treated aged hearts **(A–c)** 4 weeks after Y-CT-exos treatment. Representative images of DAPI-stained young hearts **(A–d)**, PBS-treated aged hearts **(A–e)** and Y-CT-exos-treated aged hearts **(A–f)** 4 weeks after Y-CT-exos treatment. Representative images of immunofluorescence staining with an anti-cTnT antibody in young hearts **(A–g)**, PBS-treated aged hearts **(A–h)** and Y-CT-exos-treated aged hearts **(A–i)** 4 weeks after Y-CT-exos treatment. Representative images of immunofluorescence staining with an anti-vimentin antibody in young hearts **(A–j)**, PBS-treated aged hearts **(A–k)** and Y-CT-exos-treated aged hearts **(A-l)** 4 weeks after Y-CT-exos treatment. Representative merged images of stained young hearts **(A,a–j)**, PBS-treated aged hearts **(A–b–k)** and Y-CT-exos-treated aged hearts **(A-c-l)** 4 weeks after Y-CT-exos treatment. **(B)** Comparison of the semiquantification of β-gal-positive cardiomyocytes in young hearts, PBS-treated aged hearts and Y-CT-exos-treated aged hearts 4 weeks after Y-CT-exos treatment. **(B)** Comparison of the semiquantification of β-gal-positive cardiomyocytes in young hearts, PBS-treated aged hearts and Y-CT-exos-treated aged hearts 4 weeks after Y-CT-exos treatment. **(C)** Comparison of the semiquantification of β-gal-positive cardiac fibroblasts in young hearts, PBS-treated aged hearts and Y-CT-exos-treated aged hearts 4 weeks after Y-CT-exos treatment. Red arrowheads: β-gal-positive cardiomyocytes. White arrowheads: β-gal-positive cardiac fibroblasts. **: *p* < 0.01. n = 4.

Cellular senescence plays a pivotal role in and has deleterious effects on heart aging. Therefore, the ability of Y-CT-exos to improve cellular senescence was first investigated in the present study. We performed β-gal + anti-cTnT and βgal + anti-vimentin double staining to assess senescence in cardiomyocytes and cardiac fibroblasts in the myocardium of young hearts, PBS-treated aged hearts and Y-CT-exos-treated aged hearts. The density of β-gal-positive cardiomyocytes and cardiac fibroblasts in aged hearts was significantly greater than that in young hearts, suggesting that cardiomyocytes and cardiac fibroblasts in aged hearts are senescent. In addition, the density of β-gal-positive cardiomyocytes and cardiac fibroblasts in Y-CT-exos-treated aged hearts was significantly lower than that in PBS-treated aged hearts (Figure 2). These findings suggested that Y-CT-exos treatment of aged hearts improved cardiomyocyte and noncardiomyocyte senescence.

### Y-CT-exos treatment reverses the age-related increases in the expression of the cellular senescence marker genes p21, p16, mTOR in aged hearts

**FIGURE 3:**
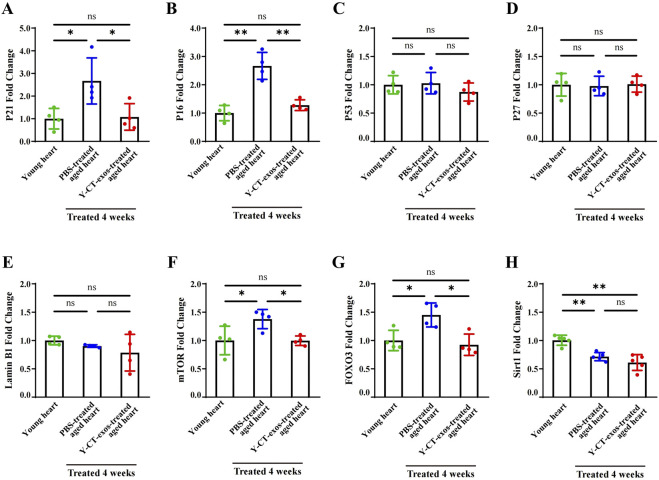
Y-CT-exos treatment reverses the aging-related increase in the expression of the cellular senescence marker genes (p21, p16, and mTOR) in aged hearts. *p21* gene **(A)**, *p16* gene **(B)**, *p53* gene **(C)**, *p27* gene **(D)**, *Lamin B1 gene* **(E)**, *mTOR gene* **(F)**, *FOXO3* gene **(G)** and *Sirt1* gene **(H)** expression in young hearts, PBS-treated aged hearts and Y-CT-exos-treated aged hearts 4 weeks after Y-CT-exos treatment. *: *p* < 0.05. **: *p* < 0.01. Ns: *p* > 0.05. N = 4.

The effects of Y-CT-exos treatment on representative marker genes of cellular senescence (p21, p16, p53, p27, Lamin B1 and mTOR) and longevity (FOXO3 and Sirt1) in aged hearts were also evaluated. Compared with those in young hearts, the age-related increases in the expression levels of the p21, p16, mTOR and FOXO3 genes and the aging-related decrease in the expression level of the Sirt1 gene were greater in PBS-treated aged hearts, suggesting that cellular senescence increased and longevity-related activity decreased in aged hearts. Importantly, we found that the expression of the p21, p16, mTOR and FOXO3 genes in Y-CT-exos-treated aged hearts was lower than that in PBS-treated aged hearts (Figure 3). The findings confirmed that Y-CT-exos reversed aging-related cellular senescence by decreasing the expression of the p21, p16, mTOR and FOXO3 genes.

### Treatment with Y-CT-exos reduces inflammation in the hearts and bodies of aged rats

**FIGURE 4:**
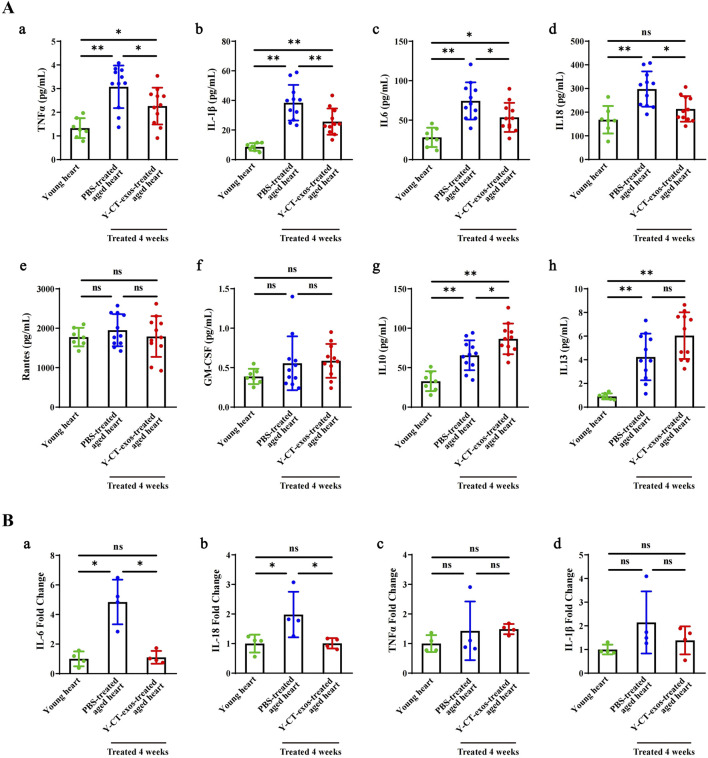
Y-CT-exos treatment decreases inflammation in the hearts and bodies of aged rats. **(A)** ELISA analysis of the serum levels of inflammatory factors (proinflammatory cytokines TNFα **(a)**, IL-1β **(b)**, IL-6 **(c)**, IL-18 **(d)**, Rantes **(e)**, and GM-CSF **(f)** and the anti-inflammatory cytokines IL-10 **(g)** and IL-13 **(h)**) in young rats, PBS-treated aged rats and Y-CT-exos-treated aged rats 4 weeks after Y-CT-exos treatment. N = 7, 11, 11. **(B)** *IL-6* gene **(a)**, *IL-18* gene **(b)**, *TNFα* gene **(c)** and *IL-1β* gene **(d)** expression in the myocardial tissue of young hearts, PBS-treated control aged hearts and Y-CT-exos-treated aged hearts 4 weeks after Y-CT-exos treatment. N = 4. *: *p* < 0.05. **: *p* < 0.01. Ns: *p* > 0.05.

Increased inflammation is a well-established phenotype of aging that is also referred to as inflammaging (López-Otín et al., 2023; Franceschi et al., 2014; Singh and Newman, 2011). Therefore, the serum levels of inflammatory factors (the proinflammatory cytokines TNFα, IL-1β, IL-6, IL-18, Rantes, and GM-CSF and the anti-inflammatory cytokines IL-10 and IL-13) in young rats, PBS-treated aged rats and Y-CT-exos-treated aged rats were compared. ELISAs of proinflammatory cytokines revealed that the serum levels of TNFα, IL1-β, IL-6 and IL-18 in the PBS-treated control aged rats were significantly greater than those in young rats, whereas the serum levels of Rantes and GM-CSF in the PBS-treated aged rats were similar to those in young rats (Figures 4Aa–f), which suggested that inflammation, which was caused mainly by increased expression of TNFα, IL1-β, IL-6 and IL-18, was increased in the bodies of aged rats compared with the bodies of young rats. Furthermore, compared with those in the PBS-treated control aged rats, the TNFα, IL-1β, IL-6 and IL-18 serum levels in the Y-CT-exos-treated aged rats were significantly lower, whereas the levels of Rantes and GM-CSF in these two groups were similar (Figures 4Aa–f). These results suggested that Y-CT-exos treatment decreases the inflammation in the bodies of aged rats by decreasing the serum levels of TNFα, IL-1β, IL6 and IL18.

ELISAs revealed that the serum levels of IL-10 and IL-13 were significantly greater in PBS-treated aged rats than in young rats (Figures 4Ag,h), suggesting that the anti-inflammatory capacity of the aged rat body, which is mediated by increased expression of IL-10 and IL-13, was greater than that in the young rat body. Importantly, the serum level of IL-10 in Y-CT-exos-treated aged rats was greater than that in PBS-treated aged rats, whereas the serum level of IL-13 in these two groups was similar (Figures 4Ag,h). These findings suggest that Y-CT-exos increase the anti-inflammatory capacity of the aged body by increasing the serum level of IL-10. Taken together, these findings revealed that Y-CT-exos treatment decreased the inflammation in the bodies of aged rats by decreasing the levels of the proinflammatory cytokines TNFα, IL-1β, IL6 and IL18 and increasing the level of the anti-inflammatory cytokine IL-10.

In addition, the anti-inflammatory effect of Y-CT-exos in the myocardium was evaluated. Compared with those in young hearts, the expression levels of IL-6 and IL-18 in PBS-treated aged hearts were greater, while the expression levels of TNFα and IL-1β in these two groups were similar (Figures 4Ba–d). Importantly, the expression levels of IL-6 and IL-18 in the Y-CT-exos-treated aged hearts were lower than those in the PBS-treated control aged hearts (Figures 4Ba–d). These results suggested that Y-CT-exos treatment decreased inflammation in aged hearts by decreasing the expression levels of the proinflammatory cytokines IL-6 and IL-18. Taken together, these findings suggest that Y-CT-exos decrease inflammation in the hearts and bodies of aged rats.

### Treatment with Y-CT-exos improves the aging-related accumulation of DNA damage in aged cardiomyocytes and decreases the level of ROS in senescent cardiomyocytes

**FIGURE 5:**
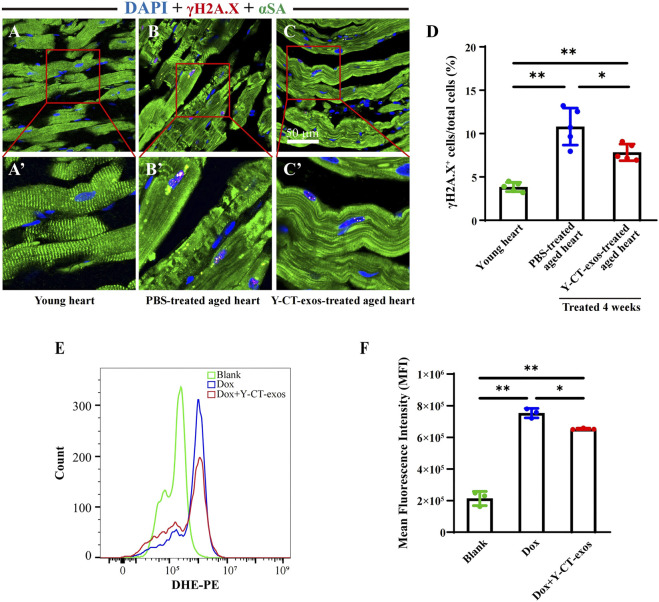
Y-CT-exos improved the aging-related accumulation of DNA and ROS damage in aged cardiomyocytes. Representative images of anti-α-SA (a marker of cardiomyocytes; green) + anti-γH2A.X (a marker of DNA damage; red) double immunofluorescence staining and DAPI counterstaining of young hearts **(A)**, PBS-treated aged hearts **(B)** and Y-CT-exos-treated aged hearts **(C)** 4 weeks after Y-CT-exos treatment. A′, B′ and C’: Magnified images of the selected area. **(D)** Comparison of the semiquantification of γH2A.X-positive cardiomyocytes in young hearts, PBS-treated aged hearts and Y-CT-exos-treated aged hearts. *: *p* < 0.05. **: *p* < 0.01. N = 5. **(E)** Flow cytometry analysis of DHE staining in *vitro* Dox-induced H9C2 cardiomyocytes in the Dox-treated group (blue), Dox-Y-CT-exos-treated group (red) and blank control group (green). **(F)** Semiquantification of the data in **(E)**. *: *p* < 0.05. **: *p* < 0.01. N = 3.

The accumulation of DNA and ROS damage in cells are among the important mechanisms of cellular senescence (Schumacher et al., 2021; Ogrodnik et al., 2019; D’adda Di Fagagna FJNRC, 2008). Therefore, the protective effect of Y-CT-exos against DNA damage in cardiomyocytes was investigated. Double immunofluorescence staining for α-SA (a marker of cardiomyocytes) and γH2A.X (a marker of DNA damage) revealed that the density of γH2A.X-positive cardiomyocytes was greater in PBS-treated aged hearts than in young hearts (Figure 5), suggesting that age-related DNA damage accumulation occurred in cardiomyocytes in aged hearts. In addition, the density of γH2A.X-positive cardiomyocytes was significantly lower in Y-CT-exos-treated aged hearts than in PBS-treated aged hearts (Figure 5). These findings suggest that Y-CT-exos protect against DNA damage in aged cardiomyocytes.

In addition, the potential protective effect of Y-CT-exos on reactive oxygen species (ROS) damage during aging was also evaluated using an *in vitro* Dox-induced H9C2 cardiomyocyte senescence model combined with ROS-DHE probe staining and flow cytometry. The fluorescence intensity of the ROS in the Dox-treated group (Dox) was significantly greater than that in the blank control group, confirming the successful establishment of the *in vitro* Dox-induced H9C2 cardiomyocyte senescence model (Figure 5). Furthermore, compared with that in the Dox group, the fluorescence intensity of ROS in the Y-CT-exos-treated Dox group (Dox-Y-CT-exos) was significantly lower. Therefore, these results suggest that Y-CT-exos protect against ROS damage in senescent cardiomyocytes.

### Treatment with Y-CT-exos treatment promotes the proliferation of cardiomyocytes in aged hearts

Increasing the proliferation potential of aged cardiomyocytes confers antiaging benefits on aging hearts. In this study, the ability of Y-CT-exos to increase the proliferation potential of cardiomyocytes was explored. Immunofluorescence staining for Ki67 (a proliferation marker) and cTnI (a cardiomyocyte marker) revealed that compared with those in young myocardia, the density of Ki67+cTnI-double-positive cardiomyocytes in aged myocardia in the PBS-treated control group significantly increased (Supplementary Figure S8). These findings suggest that increasing the proliferation potential of cardiomyocytes is necessary to combat cardiac aging. Furthermore, the density of Ki67+cTnI double-positive cardiomyocytes was greater in Y-CT-exos-treated aged myocardia than in PBS-treated control aged myocardia (Supplementary Figure S8). These results suggest that the proliferation potential of cardiomyocytes in aged hearts can increase, which can delay cardiac aging. Importantly, Y-CT-exos treatment further promoted this effect by increasing the proliferation potential of cardiomyocytes.

### Y-CT-exos alleviate age-related decreases in cardiac function and skeletal muscle motor function

Next, the effects of Y-CT-exos on improving cardiac function and skeletal muscle motor function in aged hearts and bodies were investigated. We first compared cardiac functional parameters before treatment between young hearts and aged hearts (PBS group) using echocardiography. A group of aged rats (Y-CT-exos group), which were subjected to Y-CT-exos treatment, was also analyzed to establish a baseline before treatment. Compared with those in young hearts, the ejection fraction (EF), fractional shortening (FS), mitral valve E-wave velocity (MVE) and mitral valve E-wave to A-wave ratio (MVE/A) in aged hearts were significantly lower (Supplementary Figure S9A-D), whereas the diameter, systole (diameter; s), diameter, diastole (diameter; d), volume, systole (volume; s), volume, diastole (volume; d), stroke volume and cardiac output in aged hearts were significantly greater (Supplementary Figure S9E-J1). Furthermore, the interventricular septum, systole (IVS; s), interventricular septum, diastole (IVS; d), left ventricular posterior wall, systole (LVPW; s), left interventricular posterior wall, diastole (LVPW; d), heart rate and mitral valve A-wave velocity (MVA) of aged hearts were similar to those of young hearts (Supplementary Figure S9K-P). In addition, all of the above observed parameters of aged hearts were similar to those of aged rats treated with Y-CT-exos (Supplementary Figure S9). These results revealed that cardiac function decreases and degenerative myocardial pathology occurs in aged hearts.

**FIGURE 6:**
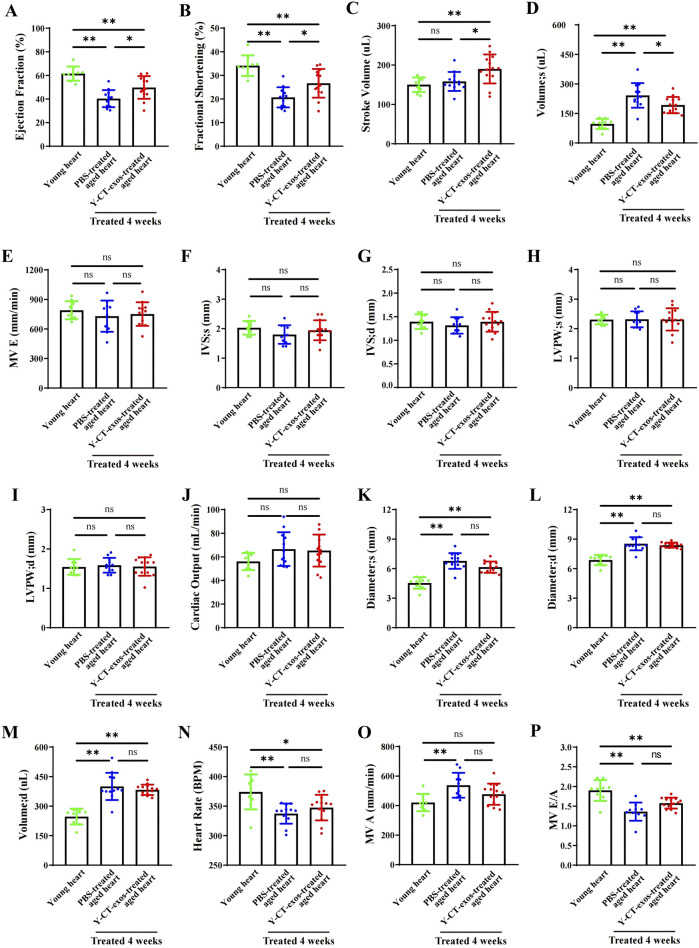
Y-CT-exos alleviated age-related decreases in cardiac function. Echocardiographic cardiac function analysis, including the ejection fraction (EF) **(A)**, fractional shortening (FS) **(B)**, stroke volume **(C)**, volume, systolic (volume; s) **(D)**, mitral valve E-wave velocity (MVE) **(E)**, interventricular septum, systole (IVS; s) **(F)**, interventricular septum, diastole (IVS; d) **(G)**, left ventricular posterior wall, systole (LVPW; s) **(H)**, left interventricular posterior wall, diastole (LVPW; d) **(I)**, cardiac output **(J)**, diameter, systole (diameter; s) **(K)**, diameter, diastole (diameter; d) **(L)**, volume, diastole (volume; d) **(M)**, heart rate **(N)**, mitral valve A-wave velocity (MVA) **(O)** and mitral valve E-wave to A-wave ratio (MVE/A) **(P)** in young hearts, PBS-treated aged hearts and Y-CT-exos-treated aged hearts 4 weeks after Y-CT-exos treatment. The EF, FS, heart rate and MVE/A of aged hearts were significantly lower than those of young hearts. The diameter; s, diameter; d, volume; s, volume; d and stroke volume of aged hearts were significantly greater than those of young hearts. Cardiac output, IVS; s, IVS; d, LVPW; s, LVPW; d and MVE of aged hearts were similar to those of young hearts. The results revealed that cardiac function decreased and that pathological myocardial degeneration occurred in aged hearts. In addition, 4 weeks after treatment, the EF, FS and stroke volume of Y-CT-exos-treated aged hearts were greater than those of PBS-treated aged hearts, whereas the volume; s of Y-CT-exos-treated aged hearts was lower than that of PBS-treated control aged hearts. These results suggested that Y-CT-exos improved the age-related decreases in left ventricular systolic and diastolic function and subsequently improved the cardiac function of aged hearts. PBS: PBS-treated aged hearts. Y-CT-exos:Y-CT-exos-treated aged hearts. *: *p* < 0.05. **: *p* < 0.01. Ns: *p* > 0.05. N = 9, 12, 13.

Next, the effect of Y-CT-exos on aged hearts was examined. We first confirmed that the cardiac function parameters of sham control hearts (thoracotomy and intramyocardial puncture only) were similar to those of PBS-treated hearts (intramyocardial injection) (*p* > 0.05) (Supplementary Figure S10), which demonstrated that the intervention itself does not have a measurable effect on cardiac function. Accordingly, in the subsequent study, the Y-CT-exos-treated aged heart group and the PBS-treated aged heart group were compared. Representative M-mode echocardiographic images of the different groups before and 4 weeks after Y-CT-exos treatment are shown in Supplementary Figure S11. Four weeks after treatment, the EF, FS and stroke volume of the Y-CT-exos-treated aged hearts were greater than those of the PBS-treated aged hearts (Figures 6A–C), whereas the volume; s of the Y-CT-exos-treated aged hearts was lower than that of the PBS-treated aged hearts (Figure 6D). In addition, the MVE, IVS; s, IVS; d, LVPW; s, LVPW; d and cardiac output of young hearts, PBS-treated control aged hearts and Y-CT-exos-treated aged hearts were similar (Figures 6E–J). In addition, the Diametr; s, Diameter; d, Volume; d, heart rate, MVA and MVE/A of the Y-CT-exos-treated aged hearts were similar to those of the PBS-treated control aged hearts (Figures 6K–P). The above results suggested that Y-CT-exos ameliorated the aging-related decreases in left ventricular systolic and diastolic function of aged hearts and subsequently improved the cardiac function.

In addition, the ability of Y-CT-exos to improve skeletal muscle motor function was investigated by assessing forelimb grip strength, endurance and maximum walking distance. Compared with those of young rats, the forelimb grip strength, endurance and maximum walking distance of PBS-treated aged rats were significantly lower. Furthermore, the forelimb grip strength of Y-CT-exos-treated aged rats was significantly greater than that of PBS-treated aged rats. However, the endurance and maximum walking distance of Y-CT-exos-treated aged rats were similar to those of PBS-treated control aged rats (Supplementary Figure S12). These findings suggested that Y-CT-exos improved the age-related decline in skeletal muscle motor function by improving forelimb grip strength.

### Treatment with Y-CT-exos reduces aging-related cardiomyocyte hypertrophy and cardiac fibrosis in the endocardium of aged hearts

**FIGURE 7:**
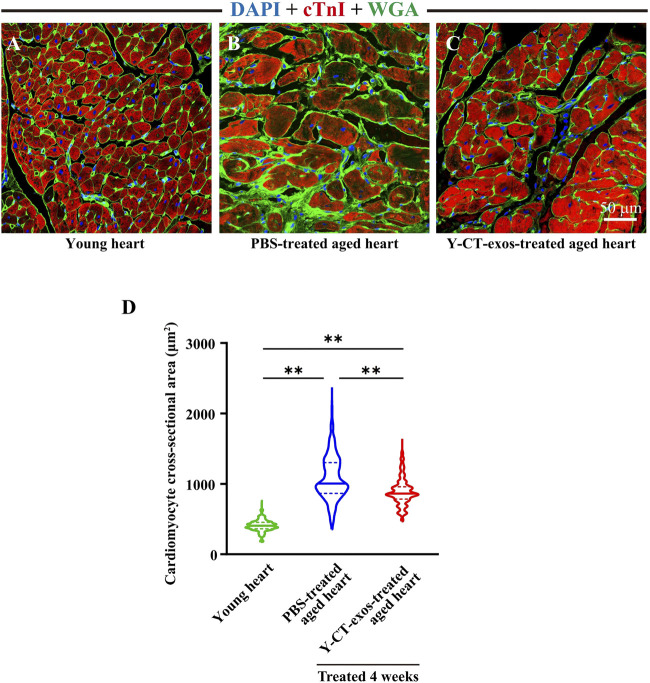
Y-CT-exos treatment reduces aging-related hypertrophy in cardiomyocytes of aged hearts. Representative images of young hearts **(A)**, PBS treated aged hearts **(B)** and Y-CT-exos-treated aged hearts **(C)** 4 weeks after Y-CT-exos treatment stained with WGA (green) and an anti-cTnI antibody (red). **(D)** Comparison of the semiquantified cardiomyocyte areas in young hearts, PBS-treated aged hearts and Y-CT-exos-treated aged hearts 4 weeks after Y-CT-exos treatment. **: *p* < 0.01. N = 5.

Cardiomyocyte hypertrophy and cardiac fibrosis are representative pathological changes associated with degeneration during cardiac aging and are major causes of heart failure (Evangelou et al., 2023; Biernacka, 2011). Accordingly, the ability of Y-CT-exos to inhibit cardiomyocyte hypertrophy was evaluated. The results of cTnI (a marker of cardiomyocytes) and WGA double staining revealed that the cardiomyocyte area in the PBS-treated control aged hearts was significantly greater than that in the young hearts, suggesting that aging-related cardiomyocyte hypertrophy increased in aged hearts. In addition, compared with those of the PBS-treated control aged hearts, the cardiomyocyte area in the Y-CT-exos-treated aged hearts was significantly lower (Figure 7). The results indicated that Y-CT-exos treatment reversed the aging-related increase in cardiomyocyte hypertrophy.

**FIGURE 8:**
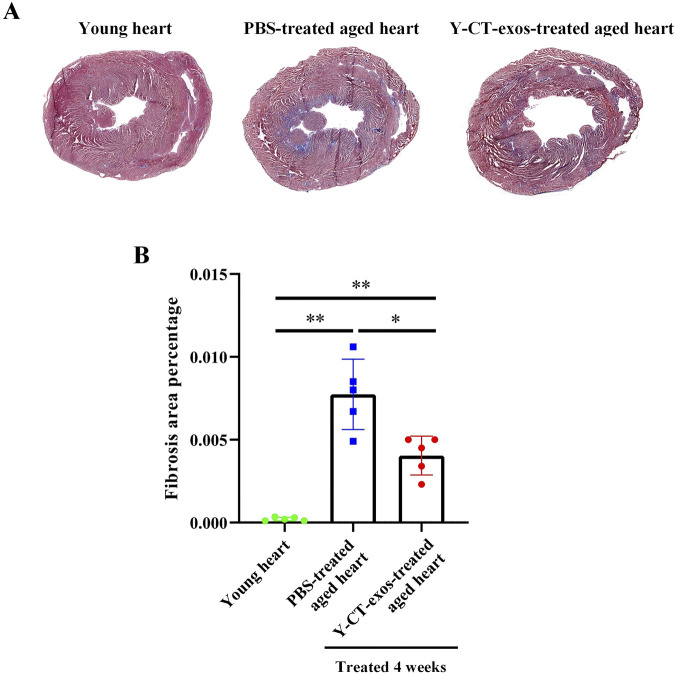
Y-CT-exos treatment decreases cardiac fibrosis in the endocardium of aged hearts. **(A)** Representative images of Masson’s trichrome staining of young hearts, PBS-treated aged hearts and Y-CT-exos-treated aged hearts 4 weeks after Y-CT-exos treatment. **(B)** Comparison of the semiquantified areas of fibrosis in young hearts, PBS-treated aged hearts and Y-CT-exos-treated aged hearts 4 weeks after YCTexos treatment. Young: Young hearts. PBS: PBS-treated aged hearts. Y-CT-exos: Y-CT-exos-treated aged hearts. *: *p* < 0.05. **: *p* < 0.01. N = 5.

In addition, the ability of Y-CT-exos to inhibit cardiac fibrosis was investigated. Masson’s trichrome staining revealed that the endocardial fibrosis area was significantly greater in the hearts of the PBS-treated control group than in those of the young group, suggesting that aging-related cardiac fibrosis increased in aged hearts. Furthermore, compared with that of the PBS-treated aged hearts, the endocardial fibrosis area in the Y-CT-exos-treated aged hearts was significantly lower (Figure 8). These results suggest that Y-CT-exos treatment reverses the aging-related increase in endocardial fibrosis.

### Transcriptome sequencing combined with IPA reveals the genes, associated pathways and interaction networks involved in the Y-CT-exos-mediated amelioration of cellular senescence, cardiac hypertrophy and inflammaging in aged hearts

**FIGURE 9:**
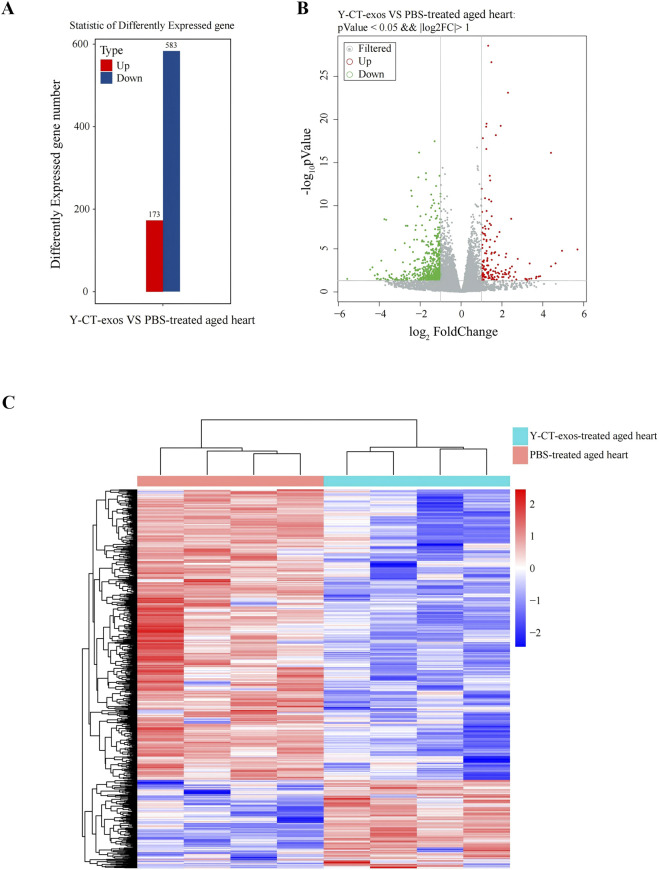
Comparison of the DEGs between Y-CT-exos-treated aged hearts and PBS-treated aged hearts identified by transcriptome sequencing and cluster analysis. **(A)** A total of 756 DEGs (*p* < 0.05, |log_2_FC|>1) were identified. Among these genes, 173 genes were upregulated and 583 genes were downregulated in Y-CT-exos-treated aged hearts compared with those in PBS-treated aged hearts. **(B)** Volcano plot of the DEGs (Y-CT-exos-treated aged hearts vs. PBS-treated aged hearts). **(C)** Cluster analysis revealed that the expression profiles of the DEGs in Y-CT-exos-treated aged hearts were significantly different from those in PBS-treated aged hearts, whereas the expression profile of each individual DEG in each group was very similar, suggesting that Y-CT-exos treatment induces changes in the gene expression profile and their function in treated aged hearts. PBS: PBS-treated aged hearts (blue). Y-CT-exos: Y-CT-exos-treated aged hearts (red).

To investigate the underlying mechanisms related to the ability of Y-CT-exos to mediate the rejuvenation of the aging cardiac phenotypes identified in the present study, a comparison of the transcriptomes of Y-CT-exos-treated aged hearts and PBS-treated aged hearts was performed. Analysis of the sequencing data revealed that the percentage of clean reads for all the samples was greater than 95% (Supplementary Table S2). In addition, principal component (PC) analysis revealed that the gene expression profile of Y-CT-exos-treated aged hearts was significantly different from that of PBS-treated aged hearts (Supplementary Figure S13). Both the results of the clean read analysis and PC analysis suggested that the quality of the sequencing data is suitable for subsequent analysis. Next, the DEGs between Y-CT-exos-treated aged hearts and PBS-treated aged hearts were analyzed. A total of 756 DEGs (*p* < 0.05, log2FC > 1) were identified. Among them, 173 genes were upregulated and 583 genes were downregulated in Y-CT-exos-treated aged hearts compared with PBS-treated aged hearts (Figures 9A,B; Supplementary Table S3). Cluster analysis revealed that the DEG profile of the Y-CT-exos-treated aged hearts was significantly different from that of the PBS-treated aged hearts, while the expression profile of each individual DEG in each group was very similar (Figure 9C), which suggested that Y-CT-exos treatment resulted in changes in the gene expression profile and function of aged hearts.

**FIGURE 10:**
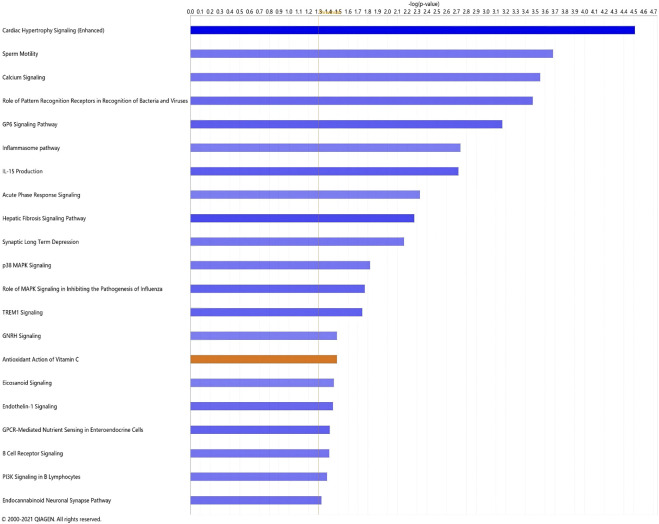
IPA revealed that Y-CT-exos suppressed the cardiac hypertrophy signaling, inflammasome pathway and p38 MAPK signaling pathway and increased the antioxidant activity of vitamin C in aged hearts. IPA of the DEGs between Y-CT-exos-treated aged hearts and PBS-treated aged hearts revealed that the suppression of the cardiac hypertrophy signaling pathway (enhanced) (listed 1), inflammasome pathway (listed 6) and p38 MAPK signaling pathway (listed 11) and increased antioxidant activity of vitamin C (listed 15) were among the top 20 enriched canonical pathways, which are related to the well-established regulatory mechanisms of cardiac hypertrophy, inflammaging and cellular senescence. Blue: Decreased pathway activity (Y-CT-exos-treated aged hearts vs. PBS-treated aged hearts). Yellow: Increased pathway activity (Y-CT-exos-treated aged hearts vs. PBS-treated aged hearts).

The identified DEGs were further subjected to IPA to identify genes related to canonical pathways and interaction networks. IPA revealed that reduced activity of the cardiac hypertrophy signaling (enhanced), inflammasome pathway and p38 MAPK signaling pathways and increased activity of the antioxidant vitamin C were predicted among the top 20 enriched canonical pathways. The reduced activity of the cardiac hypertrophy signaling (enhanced), inflammasome pathway and p38 MAPK signaling pathways was listed first (Fang et al., 2025; Kemi et al., 2000; Bitto et al., 2016), whereas the increase in the antioxidant activity of vitamin C was listed later (Abdellatif et al., 2025) (Figure 10). These findings suggest that compared with those in aged hearts treated with PBS, the cardiac hypertrophy signaling (enhanced), inflammasome and p38 MAPK signaling pathways were suppressed in aged hearts treated with Y-CT-exos, whereas the antioxidant activity of vitamin C increased.

**FIGURE 11:**
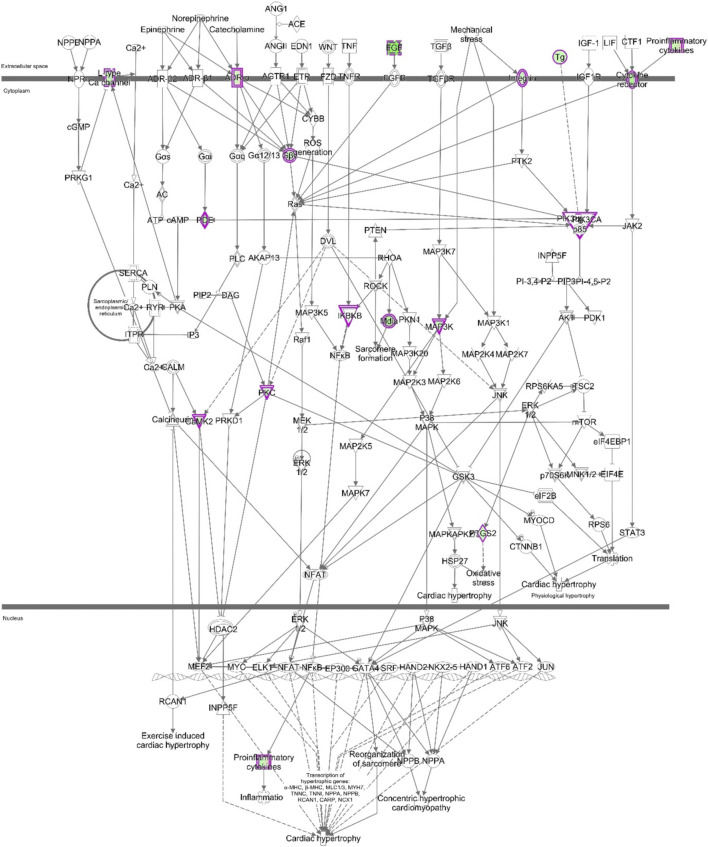
IPA revealed the genes involved in the regulation of suppressed cardiac hypertrophy signaling and their interaction networks. IPA of canonical pathways revealed that 32 DEGs (Y-CT-exos-treated aged hearts vs. PBS-treated aged hearts; 4 genes were upregulated, and 28 genes were downregulated) (Table 1) were involved in the decrease in the activity of cardiac hypertrophy signaling (enhanced). The locations of these 32 DEGs involved in cardiac hypertrophy signaling and their interactions are shown. The downregulation of the expression of 28 genes and the upregulation of the expression of 4 genes, shown by their up- and downstream interaction networks, suggest that suppressed cardiac hypertrophy signaling is an important mechanism underlying the effects of Y-CT-exos against cardiomyocyte hypertrophy. Red markers: key genes and functional molecules among the 32 DEGs. Filled in green: decreased activity.

Accordingly, the genes involved in the above pathways and their interactions, including the decreased activity of the cardiac hypertrophy signaling pathway (enhanced), were subjected to IPA. Among the 32 DEGs in the Y-CT-exos-treated aged hearts vs. PBS-treated aged hearts comparison, 4 upregulated genes and 28 downregulated genes (Table 1) were involved in the decrease in the activity of cardiac hypertrophy signaling (enhanced). The locations of the 32 DEGs involved in cardiac hypertrophy signaling and their interactions are shown in Figure 11. In fact, in this study, we demonstrated that Y-CT-exos alleviated cardiomyocyte hypertrophy in aged hearts (Figure 7). Therefore, the downregulation of the expression of 28 genes and the upregulation of the expression of 4 genes, as shown by their up- and downstream interaction network (Figure 11), suggest that the decrease in the activity of cardiac hypertrophy signaling is an important underlying molecular mechanism that mediates the effects of Y-CT-exos.

**TABLE 1:** Genes whose expression was upregulated and downregulated by Y-CT-exos and are involved in the suppression of cardiac hypertrophy signaling (enhanced), inflammasome signaling and p38 MAPK signaling and increased antioxidant activity of vitamin C.

Canonical pathways	z-score	Pathway activity	Upregulated gene	Downregulated gene
Cardiac hypertrophy signaling (enhanced)	−3.922	Decreased	Adra1d, NEWGENE621802, Pik3c2g, Tnfsf10	Adra2c, Cacna1a, Cacnb4, Camk2b, Diaph3 Faslg, Fgf14, Gng3, Ikbke, Il18, Il17re, Il18r1, Il18rap, Il1b, Il1r2, Il21r, Il22ra2, Il31ra, Itga11, Itgam, Map3k6, Map3k15, Pde4c, Pik3r5, Prkcz, Ptgs2, Tg, Tulp2
Inflammasome pathway	−2	Decreased	​	Aim2, Il18, Il1b, Nlrc4
p38 MAPK signaling	−2.121	Decreased	Hmgn1	Faslg, Il18, Il18rap, Il1b, Il1r2, Pla2g2d, Pla2g4b
Antioxidant Action of vitamin C	2.236	Increased	​	Gsto2, Ikbke, Lcat, Pla2g2d, Pla2g4b, Pla2r1, Slc23a3

**FIGURE 12:**
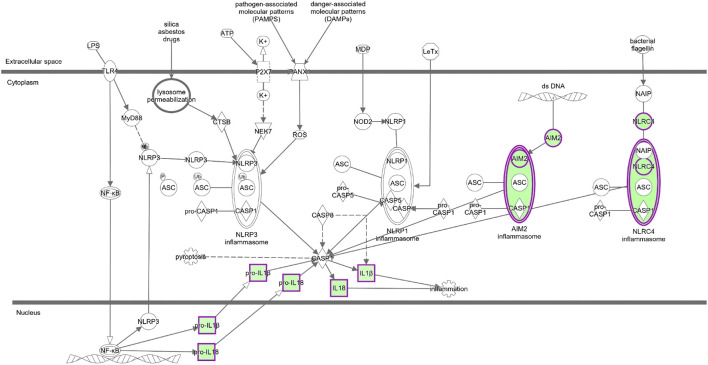
IPA revealed genes involved in the regulation of suppressed inflammasome pathway activity and their interaction networks. IPA revealed that the 4 downregulated DEGs in the Y-CT-exos-treated aged hearts vs. PBS-treated aged hearts comparison were involved in the decreased activity of the inflammasome pathway. The locations of the 4 DEGs in the inflammasome pathway and their interactions are shown. The downregulation of the expression of the 4 genes, indicated by their up- and downstream interaction networks, suggests that the decrease in the activity of the inflammasome pathway is among the important anti-inflammatory mechanisms of Y-CT-exos identified in the present study. Red markers: genes and their interaction partners among the 4 DEGs. Filled in green: decreased activity.

IPA also revealed that 4 downregulated DEGs were involved in the decreased activity of the inflammasome pathway (Table 1). The locations of these 4 DEGs in the inflammasome pathway and their interactions are shown in Figure 12. The findings reveal that the downregulation of these 4 genes, as shown in the up- and downstream interaction networks in Figure 12, suggest that the decrease in inflammasome activity is one of the important mechanisms that mediates the anti-inflammatory effects of Y-CT-exos identified in the present study.

In addition, 8 DEGs (1 whose expression was upregulated and 7 whose expression was downregulated) involved in the suppression of p38 MAPK signaling (Table 1) were identified by IPA. The locations of these 8 DEGs involved in p38 MAPK signaling and their interactions are shown in Supplementary Figure S14. Activation of p38 MAPK signaling induces cellular senescence (Iwasa et al., 2003; Freund et al., 2011); therefore, these findings suggest that the downregulation of 7 genes and the upregulation of 1 gene, as shown by their up- and downstream interaction networks in Supplementary Figure S14, result in decreased p38 MAPK signaling, which is an important mechanism underlying the antisenescence effects of Y-CT-exos in the present study.

Furthermore, IPA revealed that 7 downregulated DEGs were involved in the increased antioxidant activity of vitamin C (Table 1). The locations of the 7 DEGs in the pathway related to the antioxidant activity of vitamin C and their interactions are shown in Supplementary Figure S15. Increased vitamin C antioxidant activity plays an important role against senescence (Massip et al., 2009; Mumtaz et al., 2021); therefore, these findings suggest that the downregulation of the expression of these 7 genes, as reflected by their up- and downstream interaction networks, in Supplementary Figure S15, increases the antioxidant activity of vitamin C, which is an important mechanism underlying the antisenescence effects of Y-CT-exos documented in this study.

## Discussion

Cardiac aging is a major cause of CVD, aging-related cardiac pathological degeneration and heart failure. Currently, there is no clinical cure or treatment for cardiac aging. Although recent data have demonstrated that intervention strategies targeting cellular senescence to induce rejuvenation have great potential to delay and reverse tissue and organ aging, extensive multidisciplinary efforts are still needed to overcome the current challenges, such as identifying functional effectors and molecules and suitable ways to reverse and prevent cardiac aging. Accordingly, in the present study, we first investigated whether Y-CT-exos reversed cellular senescence in aged hearts. For this purpose, we first confirmed that after intramyocardial injection, Y-CT-exos were located in cardiomyocytes, cardiac fibroblasts and cardiac endothelial cells using *in vivo* distribution assay, and the engulfed Y-CT-exos were retained in cardiomyocytes, cardiac fibroblasts and cardiac endothelial cells for at least 24 h. These results suggest that injection of Y-CT-exos may have regulatory effects in cardiac cells such as cardiomyocytes and cardiac fibroblasts.

Indeed, we demonstrated that Y-CT-exos alleviate cardiomyocyte and cardiac fibroblasts senescence, which is supported by the findings that Y-CT-exos significantly decrease the density of β-gal (a marker of cellular senescence)-positive cardiomyocytes and cardiac fibroblasts and the expression of the cellular senescence-related genes p16, p21 and mTOR in the aged myocardium. Recent field studies have established that cellular senescence involves several underlying mechanisms, such as inflammatory aging, the accumulation of DNA damage, and the loss of cellular proliferation potential (López-Otín et al., 2023; Franceschi et al., 2014; Singh and Newman, 2011; Schumacher et al., 2021; Ogrodnik et al., 2019; D’adda Di Fagagna FJNRC, 2008; Wei and Ji, 2018; Pezone et al., 2023; Muñoz-Espín and Serrano, 2014; Adams, 2009). Therefore, the anti-inflammatory aging effect of Y-CT-exos was also evaluated. Y-CT-exos decreased inflammation in the bodies of aged rats by decreasing the serum levels of the proinflammatory cytokines TNFα, IL-1β, IL6 and IL18 and increasing the serum level of the anti-inflammatory cytokine IL-10. Furthermore, Y-CT-exos decreased inflammation in aged hearts by decreasing the expression of the proinflammatory cytokines IL-6 and IL-18. Taken together, these findings clearly suggest that Y-CT-exos decreases inflammation in the hearts and bodies of aged rats and therefore improves inflammation in aged hearts. Y-CT-exos mediated these anti-inflammatory effects in the myocardium and body unexpectedly by maintaining their level *in vivo*; in addition to injecting Y-CT-exos into the myocardium, Y-CT-exos were also injected into the tail vein 2 weeks later. These findings also suggest that Y-CT-exos can exert anti-inflammatory effects *via* focal tissue and organ injection (such as intramyocardial injection) and *via* vein injection to treat cardiac aging. In fact, the results of the *in vivo* distribution assay revealed that Y-CT-exos are distributed in the liver, spleen and lung after intramyocardial and tail vein injection. These findings suggest that Y-CT-exos have the same characteristics as exosomes from other sources and that after local injection into an organ, they can enter the circulation and accumulate in the liver, spleen and lung, similar to that after intravenous injection (Gupta et al., 2023). Given that the liver, spleen, and lung monitor and clear foreign substances and regulate immune activity, the exact molecular mechanism through which the accumulation of Y-CT-exos in these organs is related to clearance or plays a role in regulating inflammatory activity and other functions needs to be further studied. In fact, cellular senescence induces a SASP, and increasing inflammation at the tissue, organ and body levels are well-established cellular and molecular mechanisms of senescence (Muñoz-Espín and Serrano, 2014; Coppe et al., 2010; Coppé et al., 2010). Notably, Y-CT-exos decreased the expression of the proinflammatory cytokines TNFα, IL-1β, IL6 and IL18; increased the serum level of the anti-inflammatory cytokine IL-10; and decreased the expression of the proinflammatory cytokines IL-6 and IL-18 in the myocardium. All of these molecular mechanisms alleviate the inflammation in the body and myocardium of aged rats.

In addition, the accumulation of DNA damage in cells is among the recognized mechanisms of cellular senescence (Schumacher et al., 2021; Ogrodnik et al., 2019; D’adda Di Fagagna FJNRC, 2008). The ability of Y-CT-exos to protect against DNA damage was therefore investigated. Indeed, we verified that Y-CT-exos also potently decreased DNA damage in aged cardiomyocytes, which is supported by the significantly lower density of γH2A.X-positive cardiomyocytes in Y-CT-exos-treated aged hearts than in PBS-treated control aged hearts. In addition, the potential protective effect of Y-CT-exos against ROS-induced damage to cardiomyocytes was also evaluated using an *in vitro* H9C2 cardiomyocyte model of senescence induced by Dox combined with ROS-DHE staining and flow cytometry analysis. The results revealed that Y-CT-exos protect against ROS damage in senescent cardiomyocytes. Accordingly, the protective effect of Y-CT-exos on the accumulation of DNA damage and ROS in aged cardiomyocytes is another mechanism through which Y-CT-exos reverse senescence in aged cardiomyocytes. Furthermore, we revealed that the proliferation potential of cardiomyocytes can increase in aged hearts, which can delay cardiac aging. Importantly, we demonstrated that Y-CT-exos promoted the proliferation of cardiomyocytes in aged hearts, which increased resistance to the age-related loss of cardiomyocyte regeneration potential. Thus, increasing the proliferation and regeneration potential of aged cardiomyocytes and the myocardium is another mechanism through which Y-CT-exos delay cardiac aging.

On the basis of the findings that Y-CT-exos alleviate cellular senescence, inflammation, and DNA damage and ROS, increase the proliferation potential of the aged myocardium; and subsequently exert therapeutic effects on cardiac aging, we further investigated whether the identified effects against cardiac aging can also reverse and improve the aging-related decline in cardiac function and pathological changes associated with cardiac degeneration. The results of the present study confirm that Y-CT-exos improve the age-related decrease in left ventricular systolic and diastolic function and subsequently improve the function of aged hearts. These conclusion are supported by the findings that the EF, FS and stroke volume of Y-CT-exos-treated aged hearts were greater than those of PBS-treated aged hearts, whereas the volume of Y-CT-exos-treated aged hearts was smaller than that of PBS-treated control aged hearts. In addition, Y-CT-exos treatment alleviated the aging-related decline in skeletal muscle motor function, as after intramyocardial or tail vein injection of PKH26-Y-CT-exos, no fluorescence signal was detected in the fore limb or hind limb muscles. Although we did not determine how Y-CT-exos improved skeletal muscle motor function, it might be related to the improvement in cardiac function, but the underlying mechanism needs to be further investigated. Moreover, in the present study, we demonstrated that Y-CT-exos reversed the aging-related increase in cardiomyocyte hypertrophy in the myocardium and cardiac fibrosis in the endocardium. Taken together, these findings reveal that in addition to reversing cellular senescence, inflammation, and DNA and ROS damage and increasing the proliferation potential of the aged myocardium, Y-CT-exos reverse aging-related decreases in cardiac function and pathological cardiac degeneration, such as cardiomyocyte hypertrophy and cardiac fibrosis, in aged hearts and improve skeletal muscle strength and motor function. Therefore, Y-CT-exos have great therapeutic potential for development as a novel cell-free therapy to rejuvenate aged hearts and the amelioration of aging-related cardiac dysfunction and pathological cardiac degeneration, such as cardiac fibrosis and hypertrophy.

To identify the genes involved in the Y-CT-exo-mediated therapeutic effects against cardiac aging and their interaction pathways and networks, we performed transcriptome sequencing of Y-CT-exos-treated aged hearts and PBS-treated aged hearts. Analysis of the DEGs revealed that 173 genes were upregulated and 583 genes were downregulated in the hearts of Y-CT-exos-treated aged rats compared with those in the hearts of PBS-treated aged rats. The 756 identified DEGs were subjected to IPA, which is a well-accepted and powerful tool for determining the enrichment of activated and inhibited pathways and the interaction networks of related DEGs (Krämer et al., 2014; Quaade et al., 2025) according to our previously established method (Li et al., 2022). Compared with those in the hearts of PBS-treated aged rats, the activity of the cardiac hypertrophy signaling pathway, inflammasome pathway and p38 MAPK signaling pathway decreased in the hearts of Y-CT-exos-treated aged rats, whereas the antioxidant activity of vitamin C increased. These findings suggest that Y-CT-exos suppressed the inflammasome, p3 MAPK signaling and cardiac hypertrophy signaling pathways and activated activity of the vitamin C pathway in aged hearts. The findings of the present study demonstrated that Y-CT-exos reversed cellular senescence, inflammation and cardiomyocyte hypertrophy in aged hearts, and the 4 pathways identified above are known to be related to cellular senescence, inflammation and cardiomyocyte hypertrophy; therefore, the up- and downregulated genes and their up- and downstream interactions with these four pathways were further subjected to IPA.

IPA revealed that 32 genes (4 of which were upregulated and 28 of which were downregulated) were involved in the reduced activity of the cardiac hypertrophy signaling pathway (Table 1). The locations of these 32 genes in the cardiac hypertrophy signaling pathway and their up- and downstream interactions are shown in Figure 11. Therefore, the downregulation of the expression of these 28 genes and the upregulation of the expression of the 4 genes, as shown by their up- and downstream interaction networks in Figure 11, increase the activity of the cardiac hypertrophy signaling pathway, which is an important mechanism underlying therapeutic effects of Y-CT-exos in aged hearts.

IPA revealed that 4 downregulated genes (Table 1) in Y-CT-exos-treated aged hearts were involved in the decreased activity of the inflammasome pathway. The locations of these 4 genes in the inflammasome pathway and their up- and downstream interactions are shown in Figure 12. Therefore, the downregulation of the expression of these 4 genes and their up- and downstream interaction networks, as shown in Figure 12, decreases the activity of the inflammasome pathway, which is an important mechanism underlying anti-inflammatory effects of Y-CT-exos identified in the present study.

In addition, 8 genes (1 whose expression was upregulated and 7 whose expression was downregulated) were involved in the decreased p38 MAPK signaling (Table 1). The locations of these 8 genes in the p38 MAPK signaling pathway and their up- and downstream interactions are shown in Supplementary Figure S14. In addition, the 7 downregulated genes (Table 1) in Y-CT-exos-treated aged hearts were involved in increasing the antioxidant activity of vitamin C. The locations of these 7 genes in the antioxidant activity of the vitamin C pathway and their up- and downstream interactions are shown in Supplementary Figure S15. The activation of p38 MAPK signaling induces cellular senescence (Iwasa et al., 2003; Freund et al., 2011; He et al., 2020; Hongo et al., 2017; Sreedhar et al., 2016), and the activation of the antioxidant activity of vitamin C plays an important role in preventing senescence (Massip et al., 2009; Mumtaz et al., 2021; Li et al., 2016; Ma et al., 2021); therefore, these findings suggest that the downregulation of these 7 genes and the upregulation of 1 gene associated with the p38 MAPK signaling pathway, as shown by the their up- and downstream interaction networks in Supplementary Figure S14, suppress p38 MAPK signaling. Additionally, the downregulation 7 genes with the vitamin C pathway, as shown by their up- and downstream interaction networks in Supplementary Figure S15, increase the antioxidant activity of vitamin C. Both of these mechanisms are important ways through which Y-CT-exos combat cellular senescence, as determined in the present study.

In summary, the present study is the first to report that Y-CT-exos can rejuvenate aged hearts by ameliorating cardiomyocyte and cardiac fibroblast senescence, reducing DNA and ROS damage in cardiomyocytes, alleviating inflammation in the hearts and bodies of aged rats, increasing the proliferation potential of cardiomyocytes, and reversing the aging-related decreases in cardiac function, cardiomyocyte hypertrophy and cardiac fibrosis. Furthermore, the associated genes, their related pathways and up- and downstream interactions underlying the reversal of cellular senescence (inhibition of the p38 MAPK signaling pathway and activation of the antioxidant effects of vitamin C), inflammaging (inhibition of the inflammasome pathway), and cardiomyocyte hypertrophy were identified. These findings clearly suggest that Y-CT-exos, their related genes and their interactions with up- and downstream pathways have great potential for use in the development of novel cell-free therapies to attenuate cardiac aging and reverse aging-related decreases in cardiac function, cardiac fibrosis and cardiac hypertrophy. Importantly, CTs are interstitial cells and not stem cells, are associated with a low risk of tumorigenesis and naturally exhibit high cardiac affinity and compatibility. These characteristics make CTs and Y-CT-exos suitable as novel sources for the development of cell-free therapies to rejuvenate aging hearts and reverse degenerative myocardiopathy. However, owing to limitations in terms of time, aging *in vivo* models and techniques, in the present study, we were unable to identify the exact molecules involved in the identified effects or the detailed molecular regulatory mechanisms underlying the activation and inhibition of the detected pathways. These important issues, along with the mechanisms that have not yet been elucidated, will be addressed in future studies.

We recently performed miRNA sequencing of Y-CT-exos to elucidate their miRNA profile, as exosomal miRNAs are key functional effectors involved in regulating the functions of target cells. Analysis of the interactions between miRNAs and the downregulated genes in the four identified pathways (Table 1) was performed using the multiMiR R package v1.30.0 (Ru et al., 2014), miR-92a-3p was predicted to specifically target the Faslg gene which is involved in the suppression of cardiac hypertrophy signaling and p38 MAPK signaling identified in present study, and miR-92a-3p was the most abundant of the predicted miRNAs among all the downregulated genes in the four pathways identified in the present study (data not shown). The primary function of miRNAs is to downregulate the expression of their target genes, whereas the Faslg gene has been reported to regulate cardiac hypertrophy, postinfarction ventricular remodeling and heart failure (Badorff et al., 2002; Wollert et al., 2000; Li et al., 2004). The present study revealed that Y-CT-exos treatment decreased the expression of Faslg; therefore, exosomal miRNAs of Y-CT-exos, such as miR-92a-3p will be important candidates for further in-depth studies on how Y-CT-exos regulate the activity of the four pathways mentioned above and subsequently rejuvenate cardiac aging. Taken together, the novel findings of the present study provide a new source and mechanistic foundation for the development of novel strategies to design cell-free therapies to rejuvenate aging hearts and combat aging-related degenerative myocardiopathy.

## Data Availability

The datasets presented in this study can be found in online repositories. The names of the repository/repositories and accession number(s) can be found below: https://ngdc.cncb.ac.cn/gsa, GSA: CRA039489.
