# Supplementary material for: Young cardiac telocyte-derived exosomes rejuvenate aging hearts in rats
Source: Front Cell Dev Biol. 2026 Jul 9;14:1824533. doi: 10.3389/fcell.2026.1824533 (PMC13391955; doi:10.3389/fcell.2026.1824533)
Supplement: Supplementary file 2 [file Supplementaryfile1.docx]

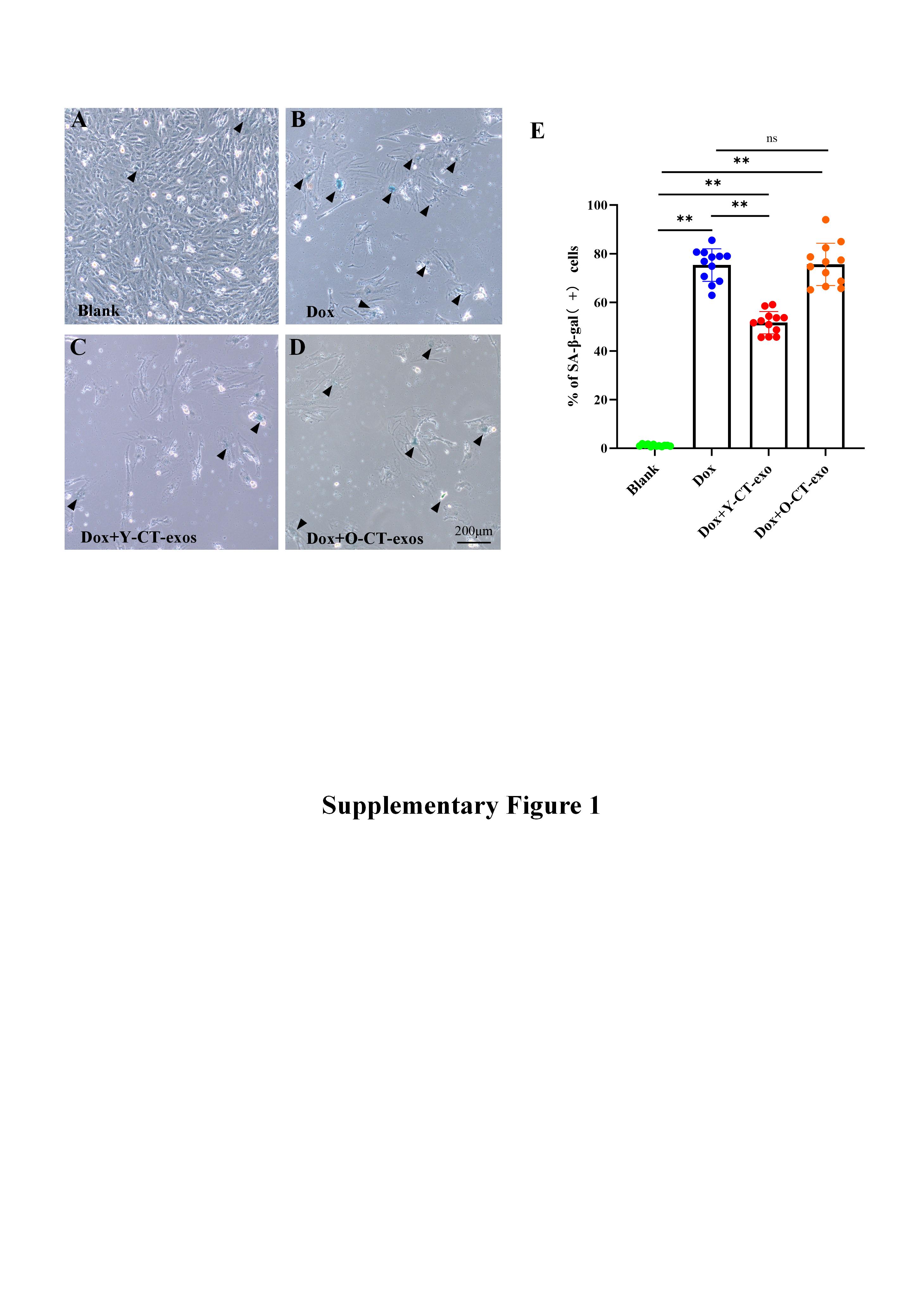


**Supplementary Figure 1: Y-CT-exos treatment alleviates senescence in cardiomyocytes, but O-CT-exos treatment does not.** Doxorubicin (Dox) was used to induce H9C2 cardiomyocyte senescence *in vitro*, and β-gal staining was performed to compare the antisenescence effects of Y-CT-exos and O-CT-exos. H9C2 cardiomyocytes were treated with Dox for 24 hours to induce senescence. Y-CT-exos and O-CT-exos were added after 24 hours of Dox treatment, and β-gal staining was performed after 96 hours of treatment. **A:** β-gal staining image of blank control H9C2 cardiomyocytes. **B:** β-gal staining image of Dox-treated H9C2 cardiomyocytes. **C:** β-gal staining image of Y-CT-exos+Dox-treated H9C2 cardiomyocytes. **D:** β-gal staining image of O-CT-exos+Dox-treated H9C2 cardiomyocytes. **E:** Semiquantification of the data in A-D. The density of β-gal-positive cardiomyocytes in the Dox-treated group (Dox) was significantly greater than that in the blank control group, which confirmed the successful induction of senescence. In addition, compared with that in the Dox-treated senescent control group, Y-CT-exos+Dox treatment significantly decreased the density of β-gal positive cardiomyocytes, while the density of β-gal positive cardiomyocytes in the O-CT-exos+Dox group was similar, which suggests that Y-CT-exos treatment alleviates cardiomyocyte senescence but O-CT-exos treatment does not. *: *p*<0.05. **: *p*<0.01. ns: *p*>0.05. n=12.


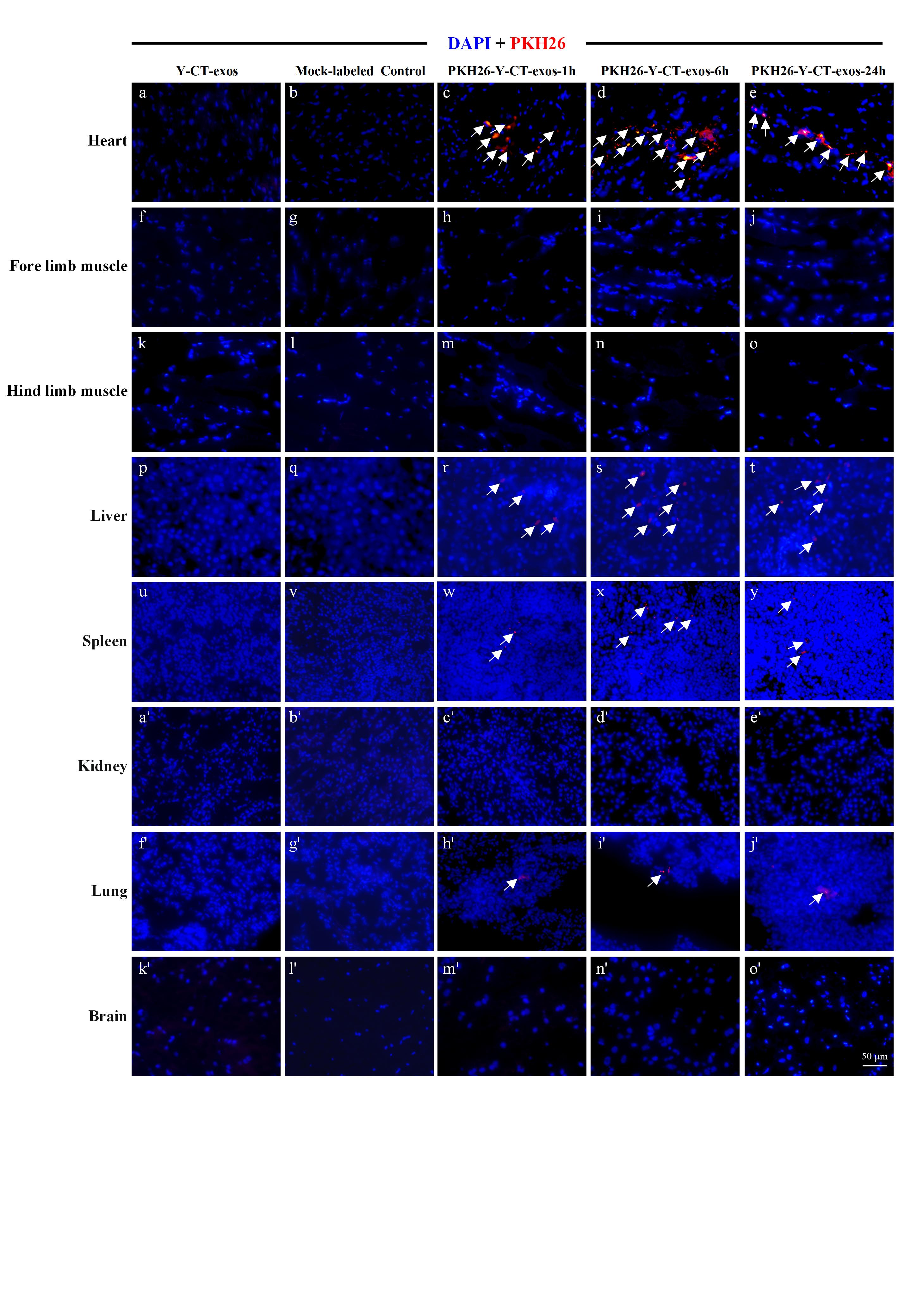


**Supplementary Figure 2: *In vivo* distribution of Y-CT-exos after intramyocardial injection.** Y-CT-exos were labeled with a PKH26 probe and administered by intramyocardial injection. The fluorescence signal (red) was detected in the cryosections of individual organs that had been counterstained with DAPI. A fluorescence signal was detected in the heart (**c–e**), liver (**r-t**), spleen (**w-y**) and lung (**h’-j’**) but not in the forelimb muscle (**h-j**), hind limb muscle (**m-o**), kidney (**c’-e’**) or brain (**m’-o’**) at 1 h, 6 h and 24 h after intramyocardial injection. Y-CT-exos: unlabeled Y-CT-exos. Mock-labeled control: PBS with PKH26, prepared identically. PKH26-Y-CT-exos-1h: PKH26-labeled Y-CT-exos 1 h after intramyocardial injection. PKH26-Y-CT-exos-6h: PKH26-labeled Y-CT-exos 6 h after intramyocardial injection. PKH26-Y-CT-exos-24h: PKH26-labeled Y-CT-exos 24 h after intramyocardial injection. n=2.


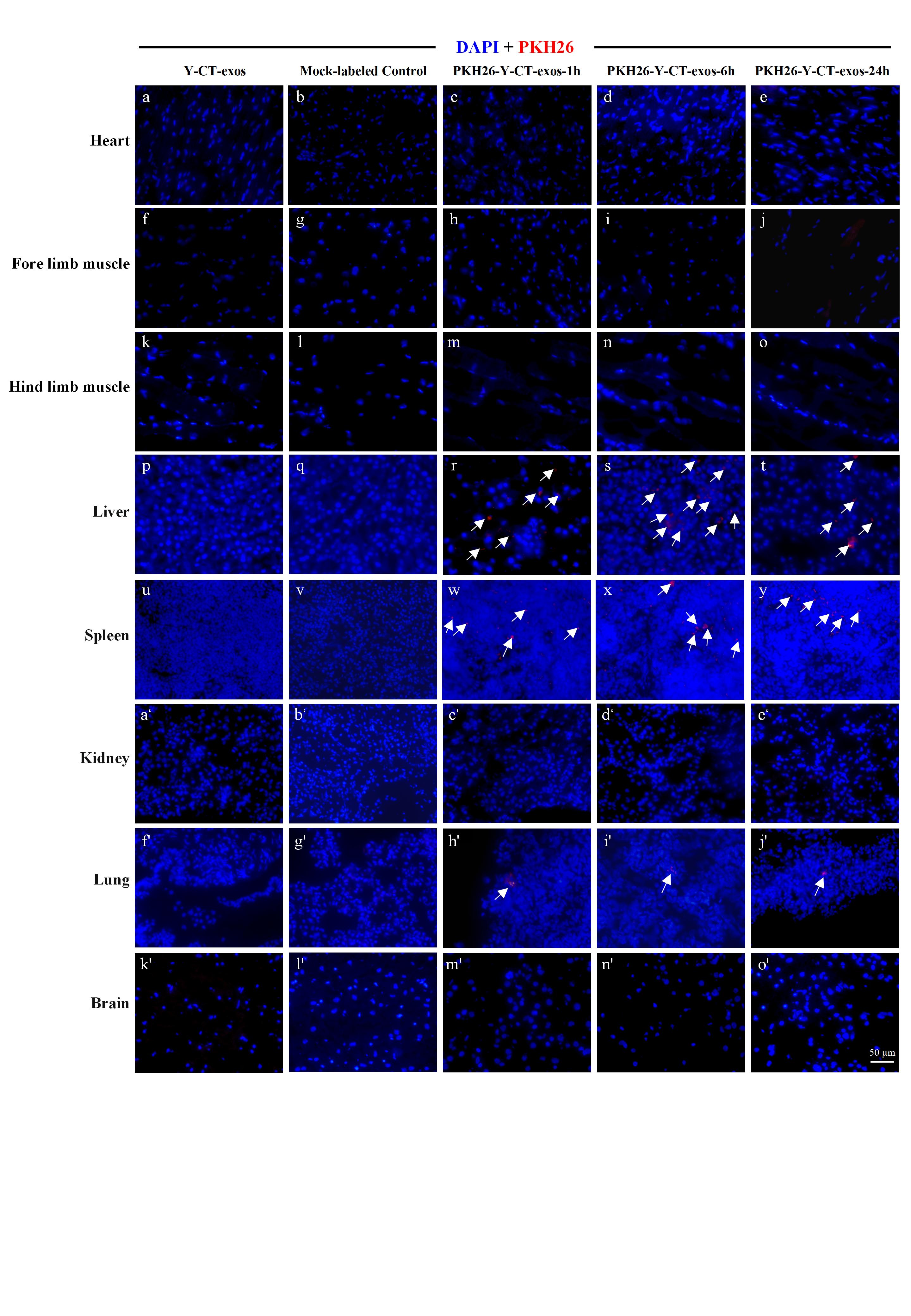


**Supplementary Figure 3: *In vivo* distribution of Y-CT-exos after tail vein injection.** Y-CT-exos were labeled with a PKH26 probe and administered by tail vein injection. The fluorescence signal (red) was detected in the cryosections of individual organs that had been counterstained with DAPI. A fluorescence signal was detected in the liver (**r-t**), spleen (**w-y**) and lung (**h’-j’**) but not in the fore limb muscle (**h-j**), hind limb muscle (**m-o**), kidney (**c’-e’**) or brain (**m’-o’**) at 1 h, 6 h and 24 h after tail vein injection. Y-CT-exos: unlabeled Y-CT-exos. Mock-labeled control: PBS with PKH26, prepared identically. PKH26-Y-CT-exos-1h: PKH26-labeled Y-CT-exos 1 h after tail vein injection. PKH26-Y-CT-exos-6h: PKH26-labeled Y-CT-exos 6 h after tail vein injection. PKH26-Y-CT-exos-24h: PKH26-labeled Y-CT-exos 24 h after tail vein injection. n=2.


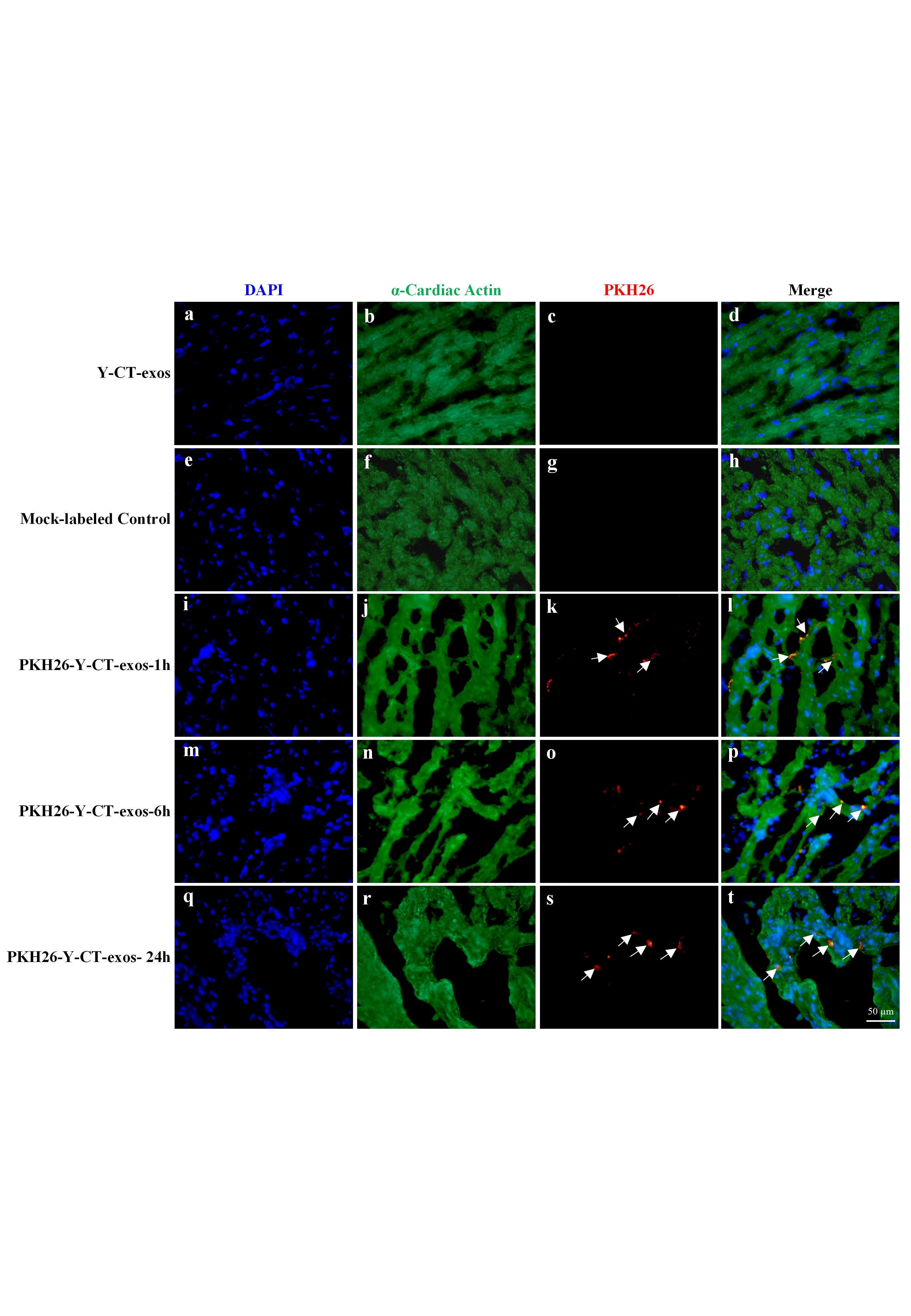


**Supplementary Figure 4: *In vivo* engulfment of Y-CT-exos by cardiomyocytes after intramyocardial injection.** *In vivo* engulfment of Y-CT-exos after intramyocardial injection of PKH26-Y-CT-exos (red) by cardiomyocytes was assessed using an anti-α-cardiac actin (a marker of cardiomyocytes) antibody for immunofluorescence staining (green). Red fluorescence signals (Y-CT-exos) were detected in cardiomyocytes at 1 h (**i-l**), 6 h (**m-p**) and 24 h (**q–t**) after intramyocardial injection. Y-CT-exos: unlabeled Y-CT-exos. Mock-labeled control: PBS with PKH26, prepared identically. PKH26-Y-CT-exos-1h: PKH26-labeled Y-CT-exos 1 h after intramyocardial injection. PKH26-Y-CT-exos-6h: PKH26-labeled Y-CT-exos 6 h after intramyocardial injection. PKH26-Y-CT-exos-24h: PKH26-labeled Y-CT-exos 24 h after intramyocardial injection. n=2.


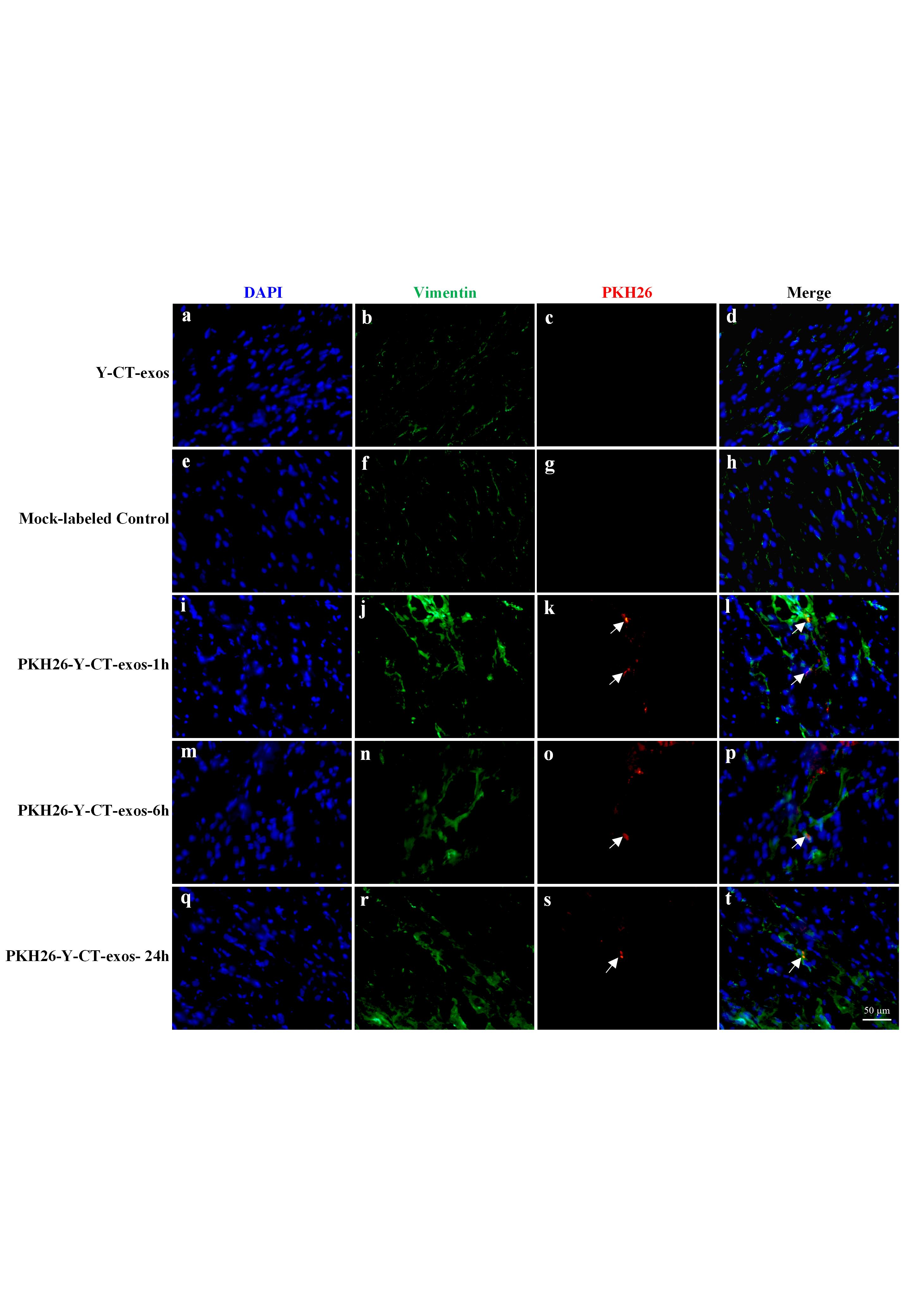


**Supplementary Figure 5: *In vivo* engulfment of Y-CT-exos by cardiac fibroblasts after intramyocardial injection.** *In vivo* engulfment of Y-CT-exos after intramyocardial injection of PKH26-Y-CT-exos (red) by cardiac fibroblasts was assessed using an anti-vimentin (a marker of cardiac fibroblasts) antibody for immunofluorescence staining (green). Red fluorescence signals (Y-CT-exos) were detected in cardiac fibroblasts at 1 h (**i–l**), 6 h (**m–p**) and 24 h **(q–t**) after intramyocardial injection. Y-CT-exos: unlabeled Y-CT-exos. Mock-labeled control: PBS with PKH26, prepared identically. PKH26-Y-CT-exos-1h: PKH26-labeled Y-CT-exos 1 h after intramyocardial injection. PKH26-Y-CT-exos-6h: PKH26-labeled Y-CT-exos 6 h after intramyocardial injection. PKH26-Y-CT-exos-24h: PKH26-labeled Y-CT-exos 24 h after intramyocardial injection. n=2.


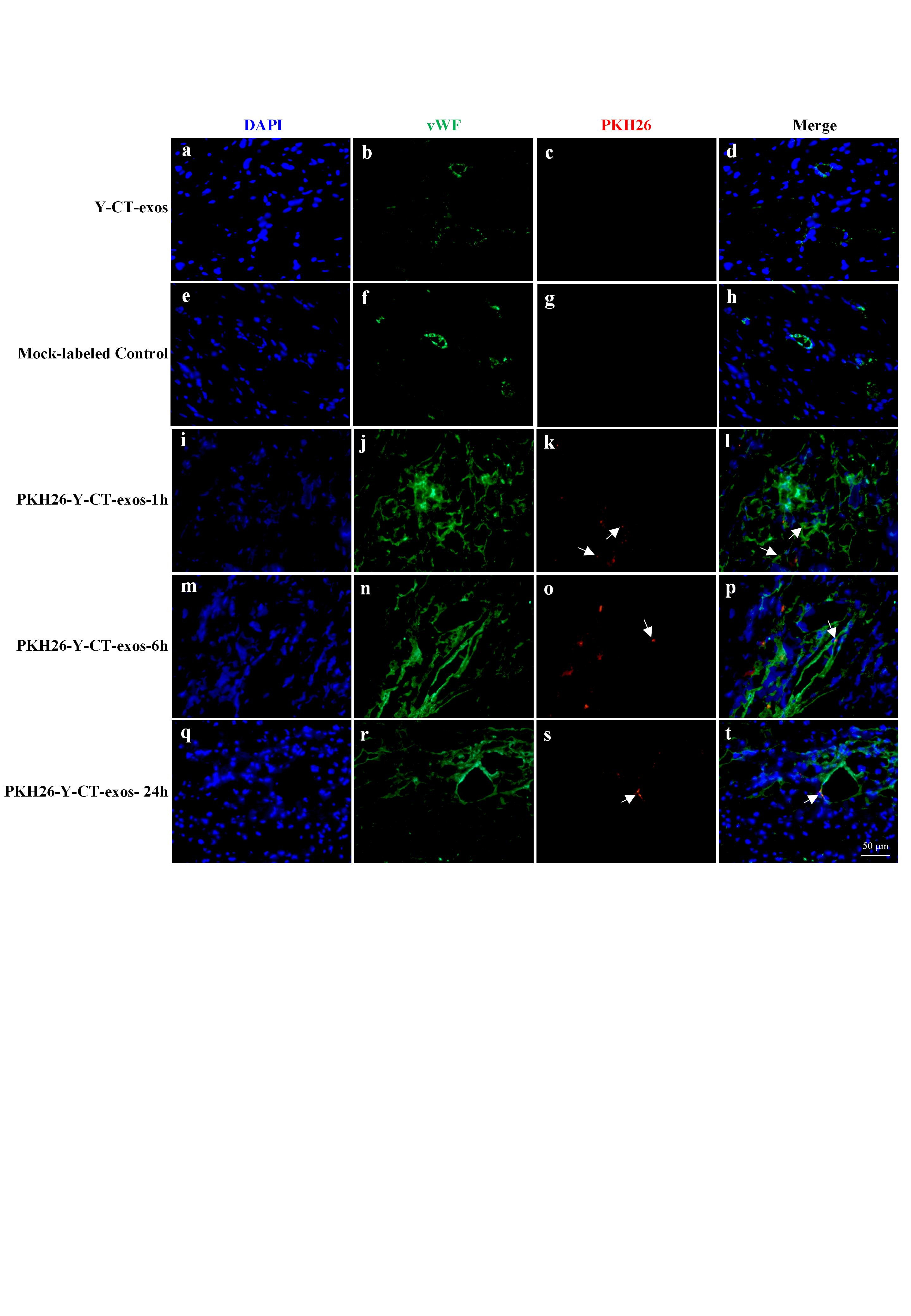


**Supplementary Figure 6: *In vivo* engulfment of Y-CT-exos by cardiac endothelial cells after intramyocardial injection.** *In vivo* engulfment of Y-CT-exos after intramyocardial injection of PKH26-Y-CT-exos (red) by cardiac endothelial cells was assessed using an anti-vWF (a marker of endothelial cells) antibody for immunofluorescence staining (green). Red fluorescence signals (Y-CT-exos) were detected in cardiac endothelial cells at 1 h (**i–l**), 6 h (**m–p**) and 24 h **(q–t**) after intramyocardial injection. Y-CT-exos: unlabeled Y-CT-exos. Mock-labeled control: PBS with PKH26, prepared identically. PKH26-Y-CT-exos-1h: PKH26-labeled Y-CT-exos 1 h after intramyocardial injection. PKH26-Y-CT-exos-6h: PKH26-labeled Y-CT-exos 6 h after intramyocardial injection. PKH26-Y-CT-exos-24h: PKH26-labeled Y-CT-exos 24 h after intramyocardial injection. n=2.


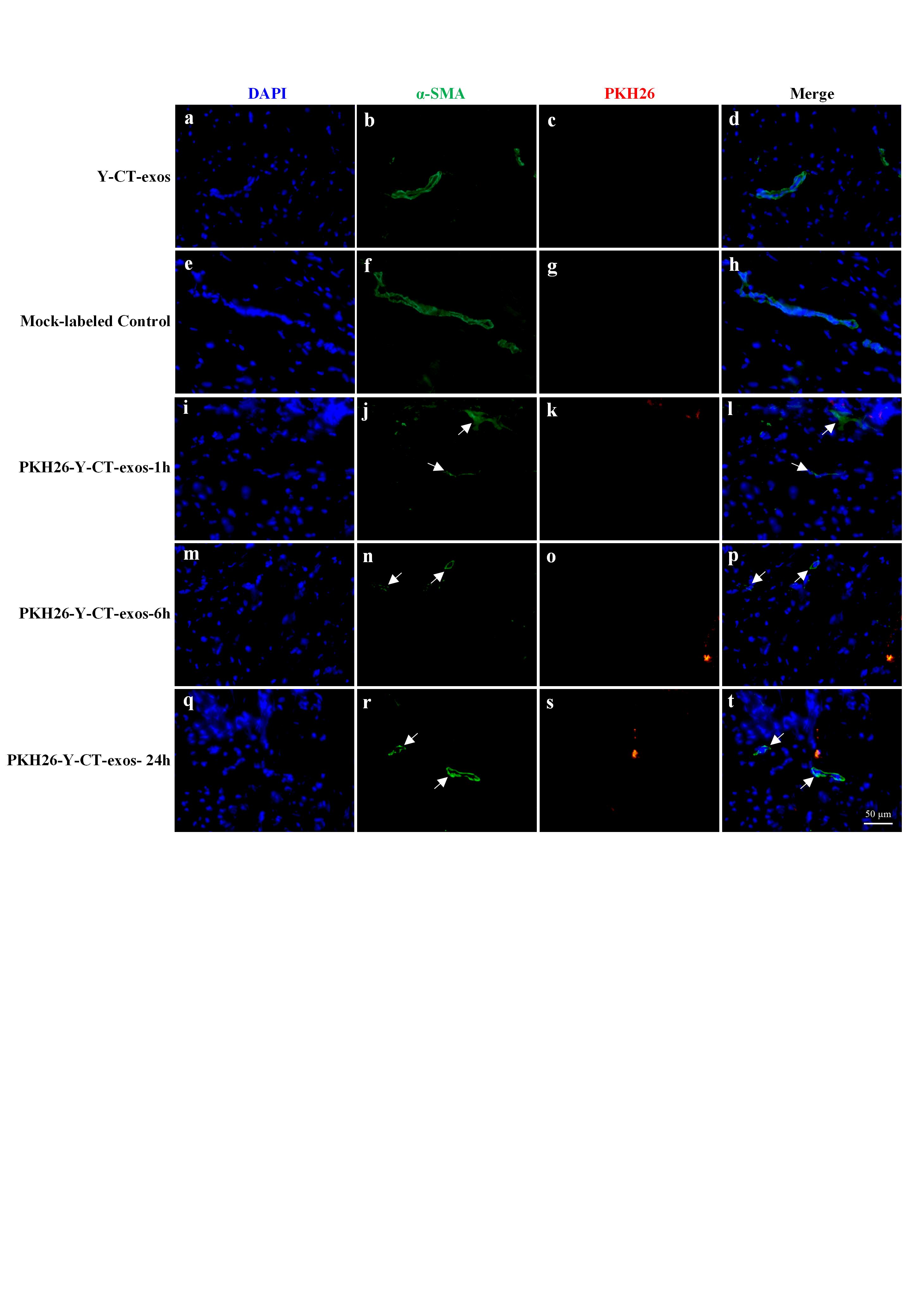


**Supplementary Figure 7: *In vivo* engulfment of Y-CT-exos by smooth muscle cells after intramyocardial injection.** *In vivo* engulfment of Y-CT-exos after intramyocardial injection of PKH26-Y-CT-exos (red) by smooth muscle cells was assessed using an anti-α-SMA (a marker of endothelial cells) antibody for immunofluorescence staining (green). Red fluorescence signals (Y-CT-exos) were detected in smooth muscle cells at 1 h (**i–l**), 6 h (**m–p**) and 24 h **(q–t**) after intramyocardial injection. Y-CT-exos: unlabeled Y-CT-exos. Mock-labeled control: PBS with PKH26, prepared identically. PKH26-Y-CT-exos-1h: PKH26-labeled Y-CT-exos 1 h after intramyocardial injection. PKH26-Y-CT-exos-6h: PKH26-labeled Y-CT-exos 6 h after intramyocardial injection. PKH26-Y-CT-exos-24h: PKH26-labeled Y-CT-exos 24 h after intramyocardial injection. n=2.


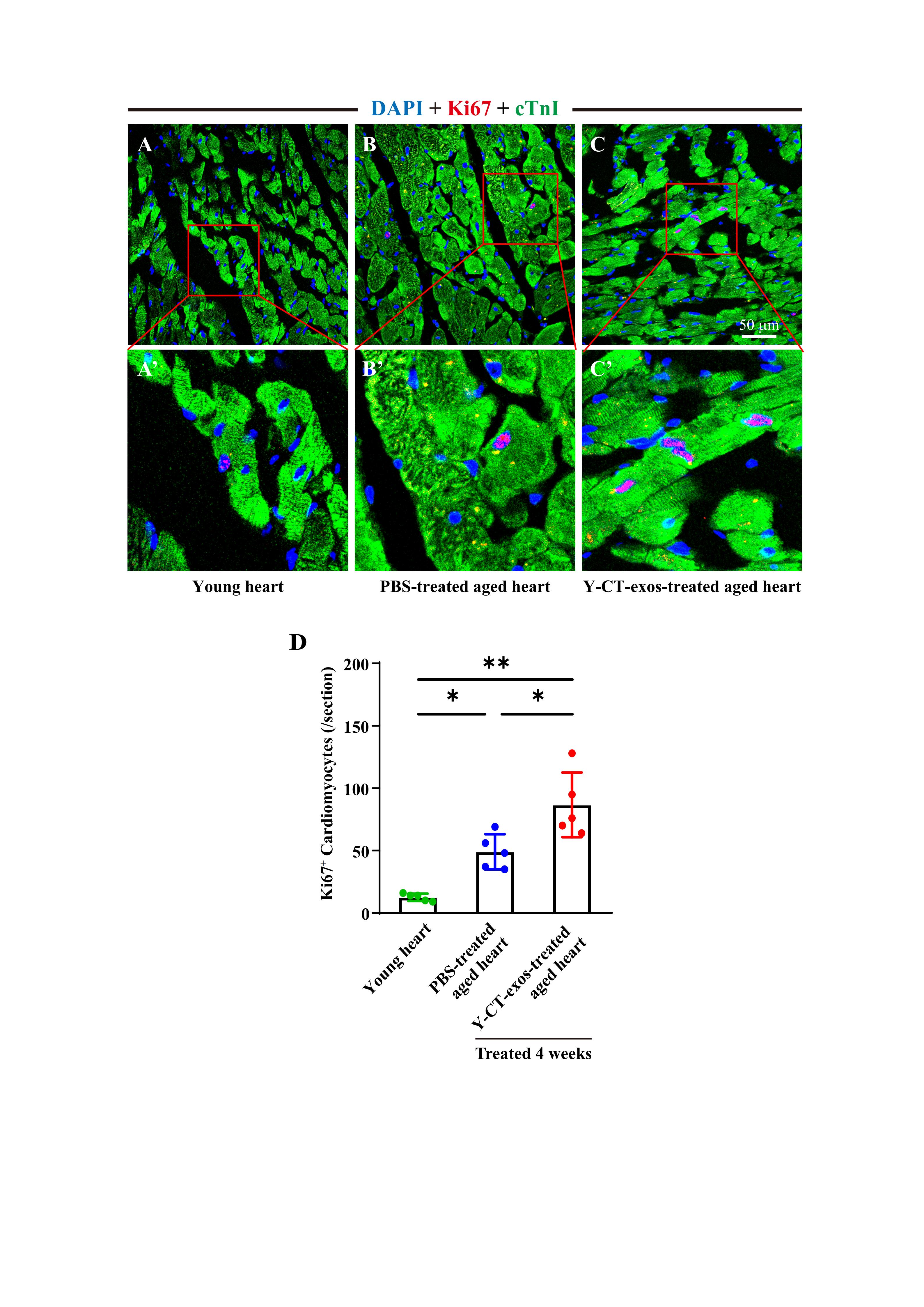


**Supplementary Figure 8: Y-CT-exos treatment increases the proliferation of cardiomyocytes in aged hearts.** Representative images of immunofluorescence staining for Ki67 (a proliferation marker) + cTnI (a cardiomyocyte marker) and DAPI counterstaining of young hearts (**A**), PBS-treated aged hearts (**B**) and Y-CT-exos-treated aged hearts (**C**) after 4 weeks of Y-CT-exos treatment. A’, B’ and C’: Magnified image of the selected area. **D:** Comparison of the semiquantified Ki67-positive cardiomyocytes in young hearts, PBS-treated aged hearts and Y-CT-exos-treated aged hearts. *: *p*<0.05. **: *p*<0.01. ns: *p*>0.05. n=5.


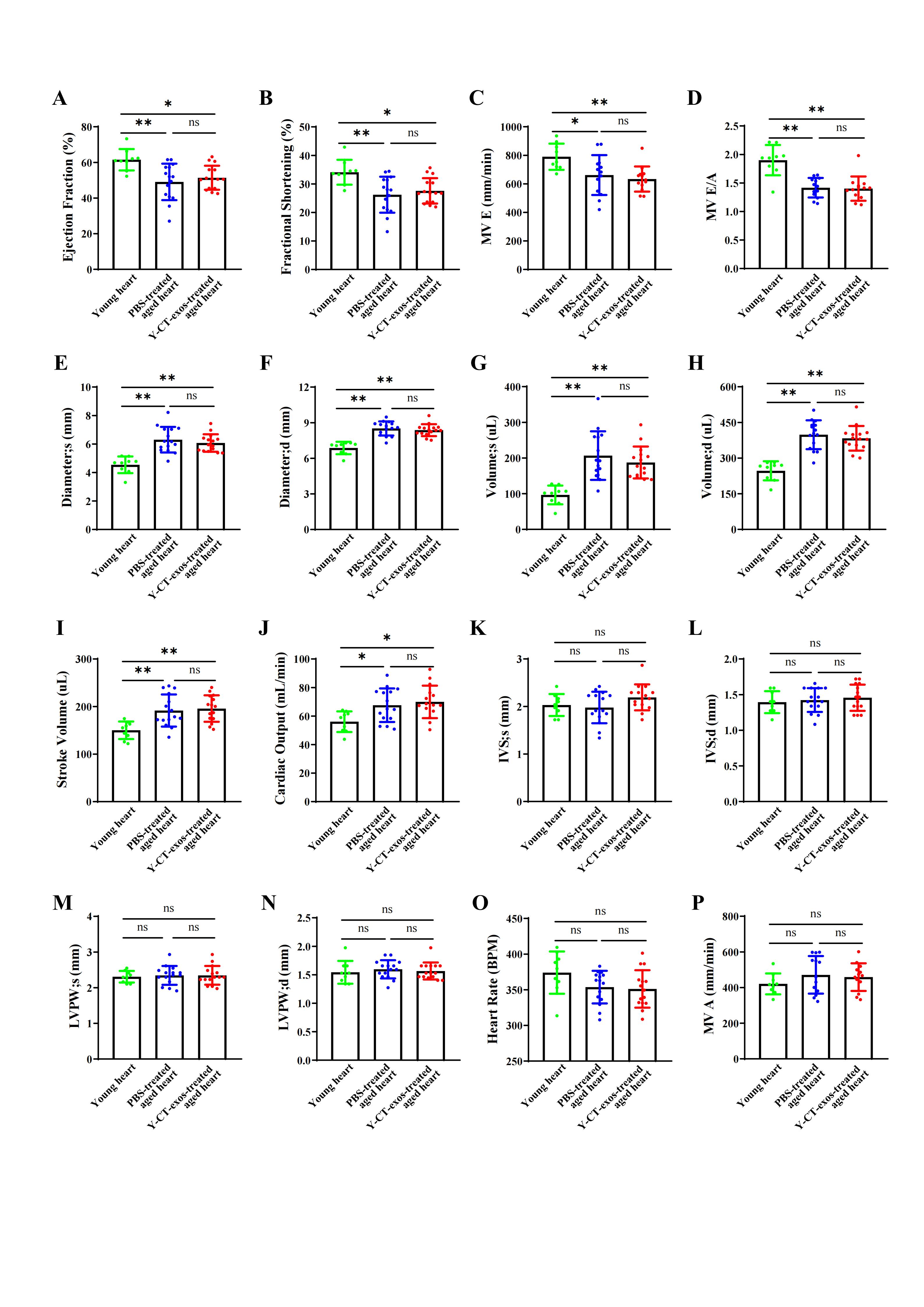


**Supplementary Figure 9: Echocardiography demonstrated that decreases in cardiac function and degenerative myocardial pathology occur in aged hearts.** To investigate the beneficial effect of Y-CT-exos on improving cardiac function in aged hearts, we first compared the cardiac functional parameters before treatment between young hearts and aged hearts (PBS group) using echocardiography. These parameters were also evaluated in a group of aged rats (Y-CT-exos group), which were subjected to Y-CT-exos treatment to establish a baseline before treatment. Echocardiography cardiac function analysis of the parameters EF (**A**), FS (**B**), MVE (**C**), MVE/A (**D**), diameter;s (**E**), diameter;d (**F**), volume;s (**G**), volume;d (**H**), stroke volume (**I**), cardiac output (**J**), IVS;s (**K**), IVS;d (**L**), LVPW;s (**M**), LVPW;d (**N**), heart rate (**O**) and MVA (**P**) in young hearts, PBS-treated aged hearts and aged hearts before Y-CT-exos treatment were analyzed. EF, FS, MVE and MVE/A were significantly lower in aged hearts than in young hearts. The diameter;s, diameter;d, volume;s, volume;d and stroke volume of aged hearts were significantly greater than those of young hearts. Cardiac output, iIVS;s, IVS;d, LVPW;s, LVPW;d, heart rate and MVA of aged hearts were similar to those of young hearts. In addition, all of the above parameters were similar for aged rats treated with PBS or Y-CT-exos. The results revealed that a decrease in cardiac function and pathological myocardial degeneration occurred in aged hearts. *: *p*<0.05. **: *p*<0.01. ns: *p*>0.05. n=9, 12, 13.


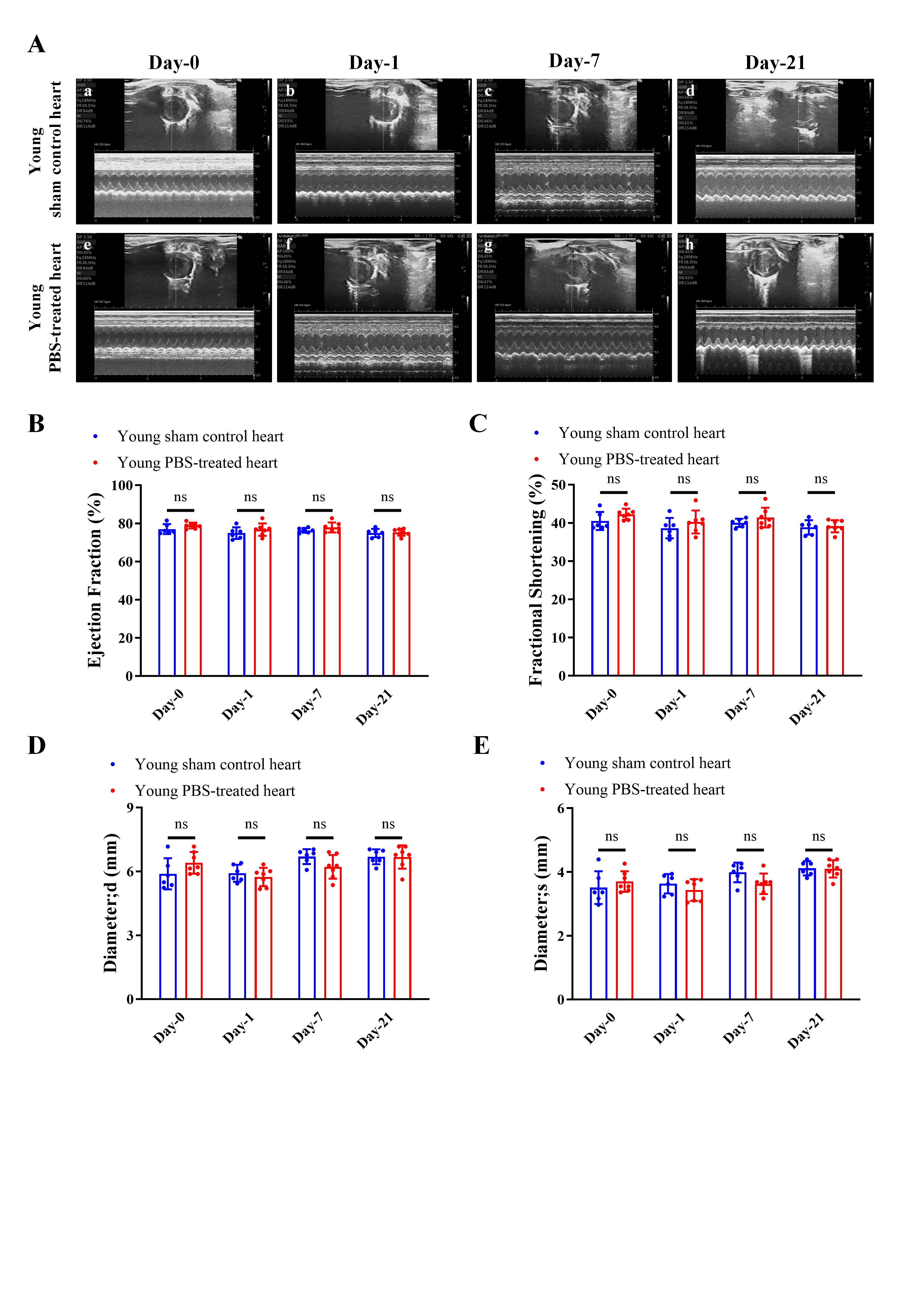


**Supplementary Figure 10:** **The cardiac function of sham control hearts was similar to that of PBS-treated hearts.** The cardiac function of young sham control hearts (thoracotomy and intramyocardial puncture only) and young PBS-treated hearts (intramyocardial injection) was compared by echocardiography on Days 0, 1, 7 and 21 after treatment. Representative surfaces measured by echocardiography on Days 0 (**A-a**), 1 (**A-b**), 7 (**A-c**) and 21 (**A-d**) in the sham group and on Days 0 (**A-e**), 1 (**A-f**), 7 (**A-g**) and 21 (**A-h**) in the PBS-treated group. **B:** Semiquantification of the ejection fraction on Days 0, 1, 7 and 21. **C:** Semiquantification of fractional shortening on Days 0, 1, 7 and 21. **D:** Semiquantification of diameter, diastole (diameter;d) on Days 0, 1, 7 and 21. **E:** Semiquantification of diameter, diastole (diameter;d) on Days 0, 1, 7 and 21. The cardiac function parameters of sham control hearts (thoracotomy and intramyocardial puncture only) were similar to those of PBS-treated hearts (intramyocardial injection) (*p*>0.05), which demonstrated that the intervention itself does not have a measurable effect on cardiac function. n=6, 7.


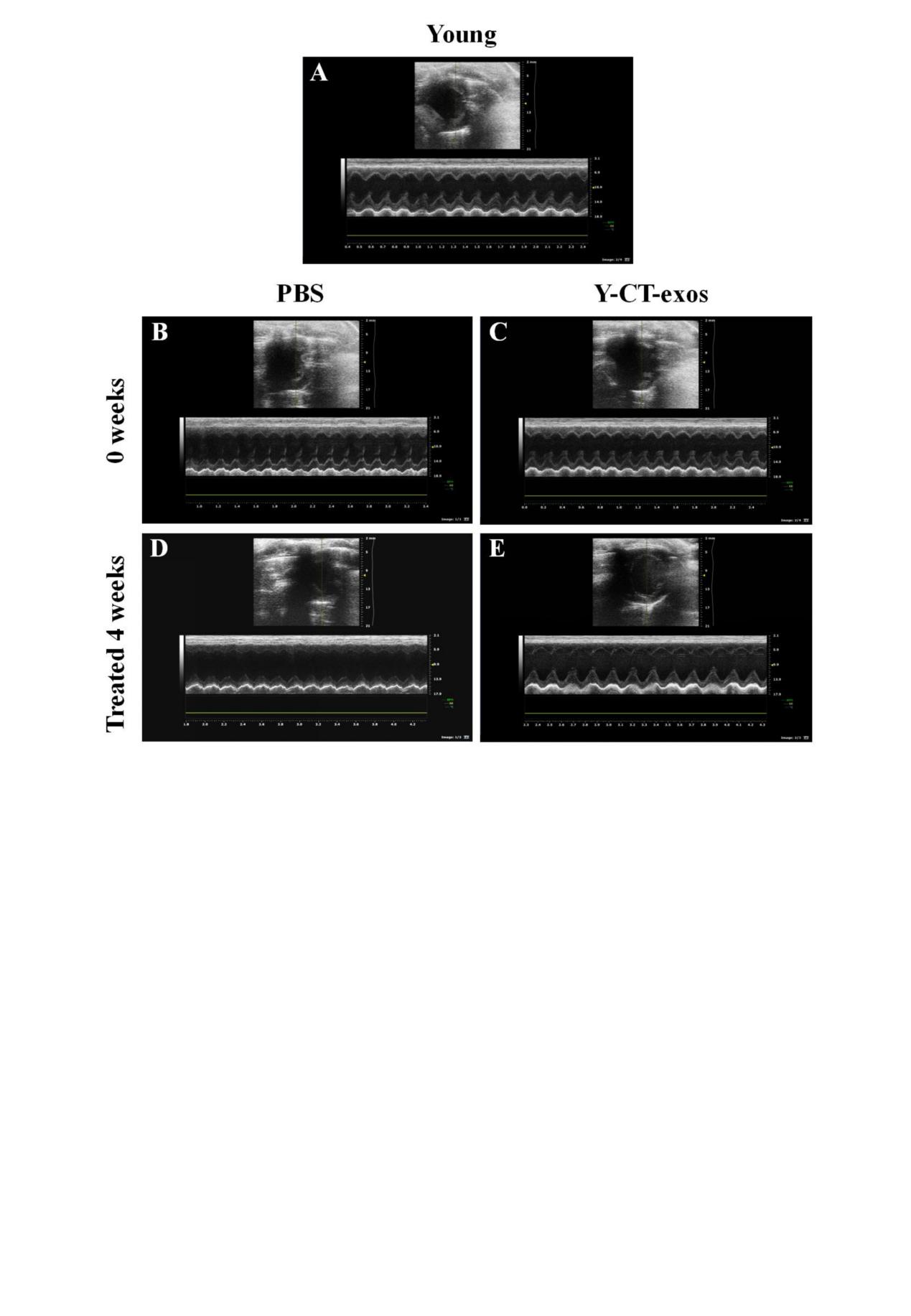


**Supplementary Figure 11: Representative echocardiography measured surfaces. A:** Young hearts. B: Aged hearts before PBS treatment. C: Aged hearts before Y-CT-exos treatment. D: Aged hearts after 4 weeks of PBS treatment. E: Aged hearts after 4 weeks of Y-CT-exos treatment.


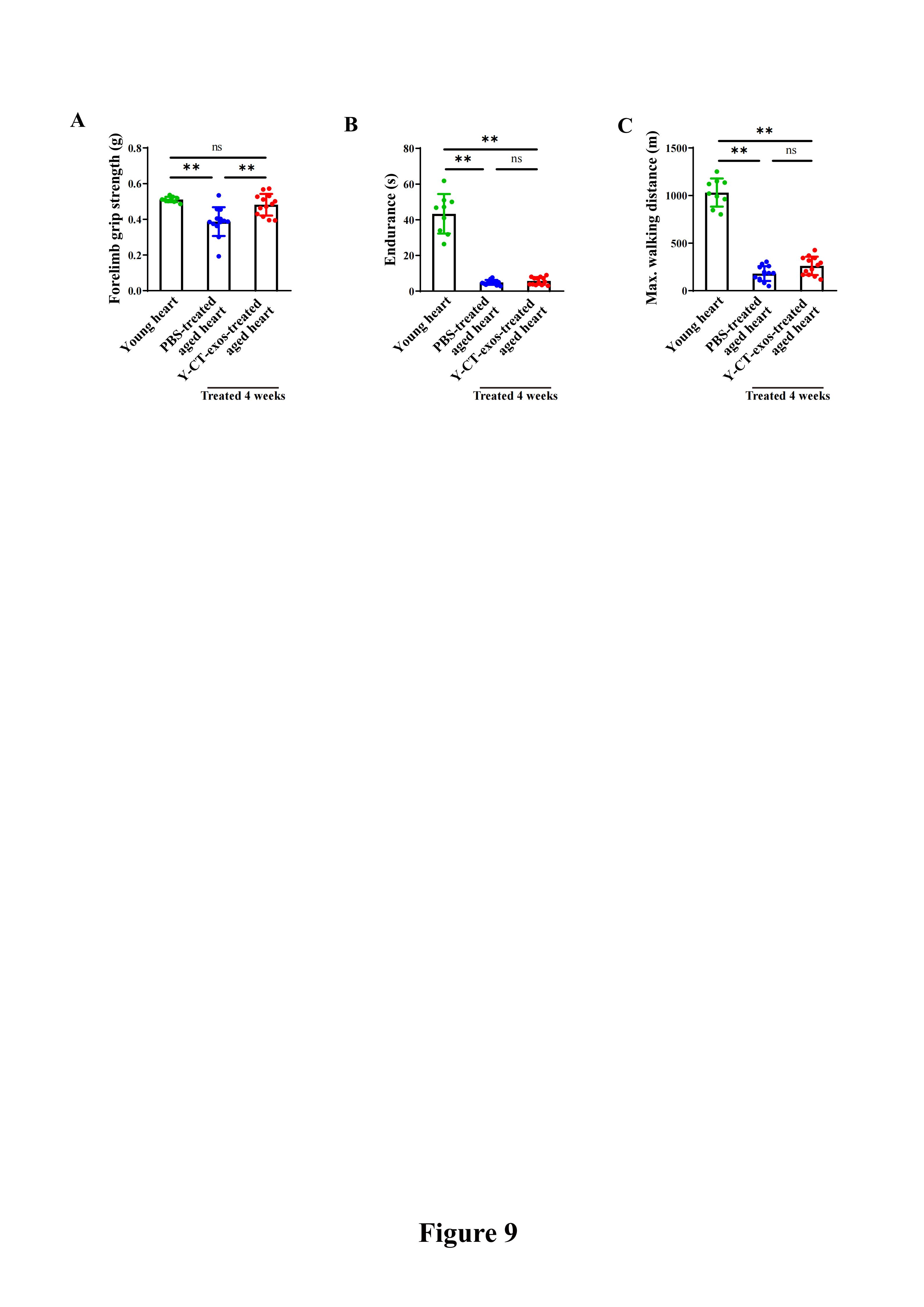


**Supplementary Figure 12: Y-CT-exos improve the age-related decrease in skeletal muscle motor function.** The ability of Y-CT-exos treatment to improve skeletal muscle motor function was investigated by assessing forelimb grip strength (A), endurance (B) and maximum walking distance (C) in young rats, PBS-treated aged rats and Y-CT-exos-treated rats after 4 weeks of Y-CT-exos treatment. Compared with those of young rats, the forelimb grip strength, endurance and maximum walking distance of PBS-treated aged rats were significantly lower. Furthermore, the forelimb grip strength of the Y-CT-exos-treated aged rats was significantly greater than that of the PBS-treated control aged rats. These results suggested that Y-CT-exos improved the aging-related decline in skeletal muscle motor function by improving forelimb grip strength. **: p<0.01. ns: *p*>0.05. n=9, 13, 13.

**
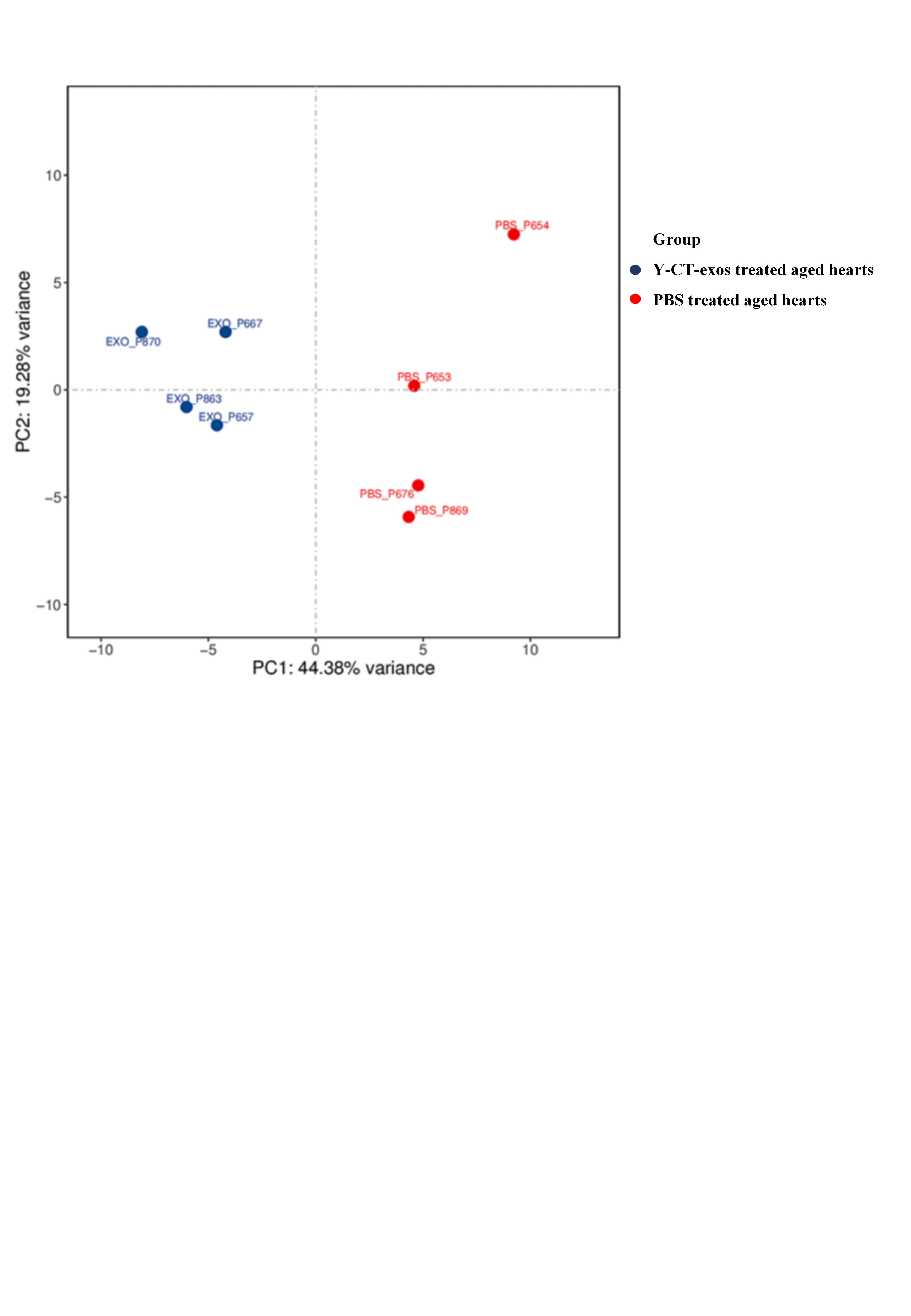
**

**Supplementary Figure 13: Principal component analysis of the transcriptome sequencing data of Y-CT-exos-treated aged hearts and PBS-treated aged hearts.** Principal component (PC) analysis revealed that the gene expression profile of Y-CT-exos-treated aged hearts was significantly different from that of PBS-treated aged hearts.


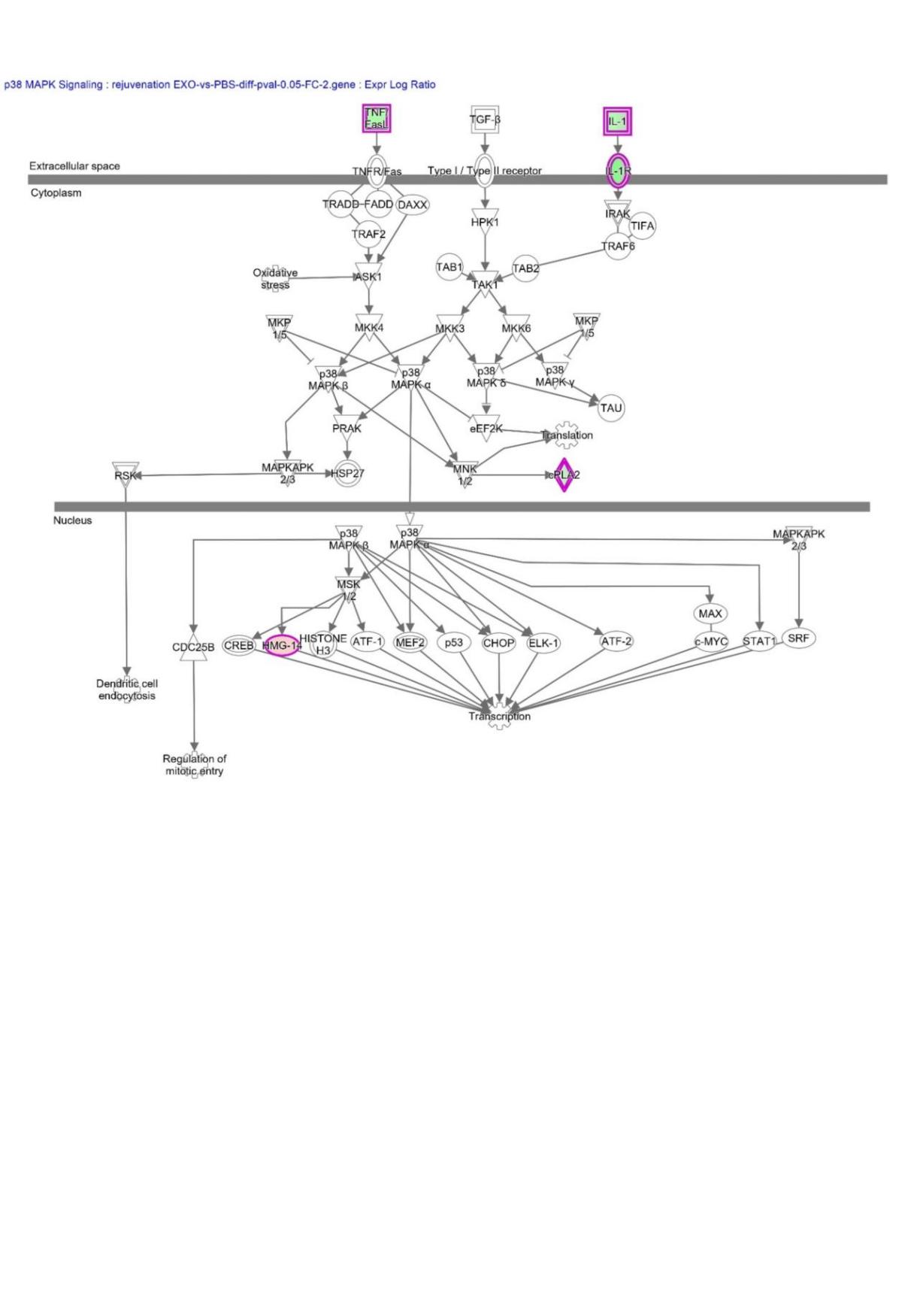


**Supplementary Figure 14: IPA revealed genes and their associated interaction networks that are involved in the regulation of decreased p38 MAPK signaling activity.** IPA of canonical pathways revealed that 8 DEGs (1 gene whose expression was upregulated and 7 genes whose expression was downregulated) were involved in the decreased activity of p38 MAPK signaling. The locations of the 8 DEGs involved in p38 MAPK signaling and their interactions are shown. These findings suggested that the downregulation of 7 genes and the upregulation of 1 gene, *via* their up- and downstream interaction network, resulted in a decrease in the activity of p38 MAPK signaling and is an important underlying antisenescence molecular mechanism of Y-CT-exos determined in the present study. Red markers: key genes and functional molecules included in the 8 DEGs. Filled in green: decreased activity. Filled in red: increased activity.


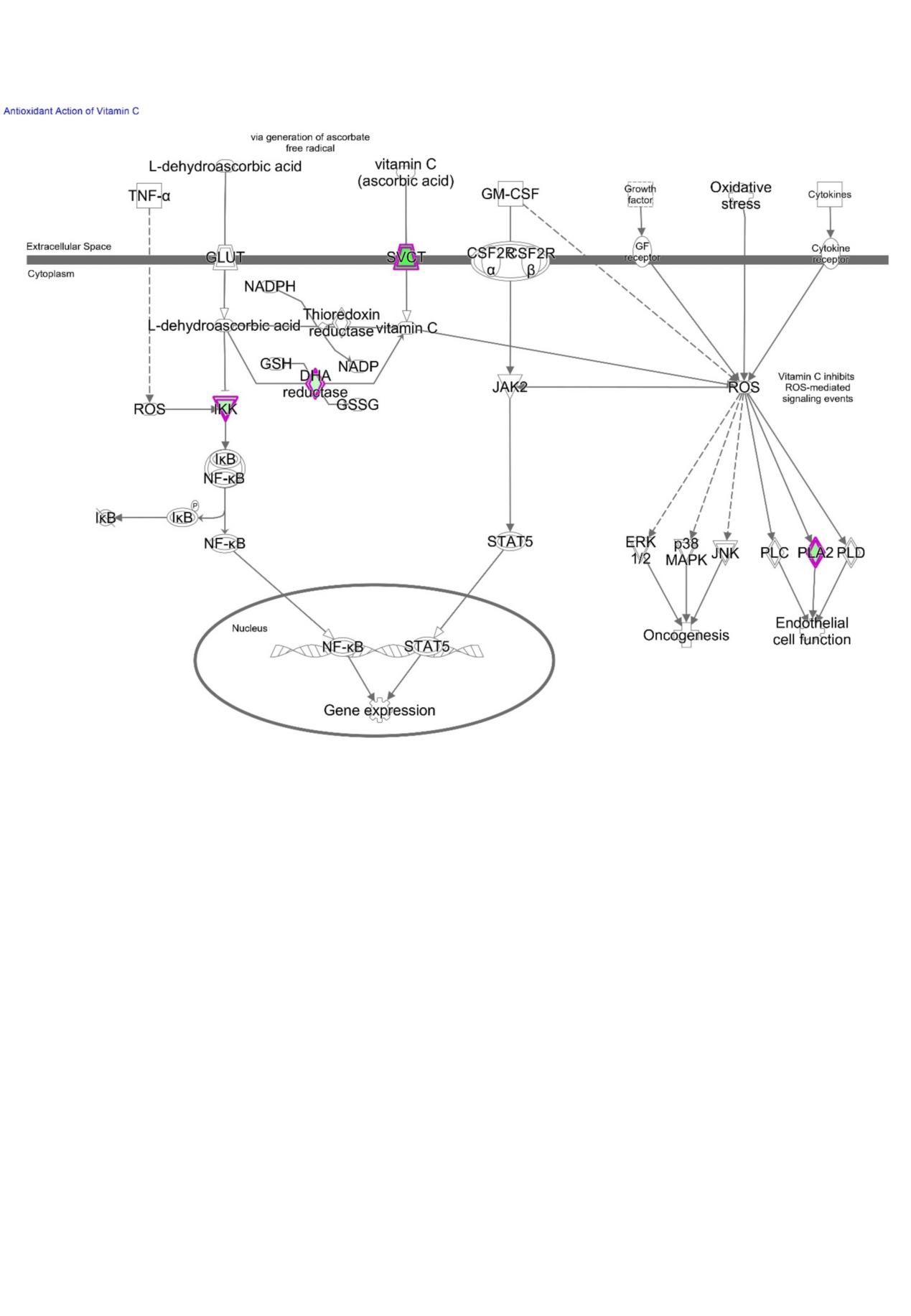


**Supplementary Figure 15: IPA revealed genes and their interaction networks that are involved in the regulation of increased activity of the vitamin C pathway.** IPA of canonical pathways revealed that 7 downregulated DEGs were involved in the increased antioxidant activity of vitamin C. The locations of the 7 DEGs in the antioxidant action of the vitamin C regulation pathway and their interactions are shown. The downregulation of the expression of the 7 genes, *via* their up- and downstream interaction network, suggests that the increase in the activity of the antioxidant activity of vitamin C is an important underlying antisenescence molecular mechanism of Y-CT-exos documented in this study. Red markers: key genes and functional molecules included in the 7 DEGs. Filled in green: decreased activity.
