# Supplementary material for: Young cardiac telocyte-derived exosomes rejuvenate aging hearts in rats
Source: Front Cell Dev Biol. 2026 Jul 9;14:1824533. doi: 10.3389/fcell.2026.1824533 (PMC13391955; doi:10.3389/fcell.2026.1824533)
Supplement: Supplementary file 3 [file Table1.docx]

**Supplementary Table 1: Sequences of q-PCR primers**

| **Primer** | **Primer Sequence** | **Product Length (bp)** |
| --- | --- | --- |
| GAPDH-F | AGACAGCCGCATCTTCTTGT | 207 |
| GAPDH-R | CTTGCCGTGGGTAGAGTCAT |  |
| P16-F | GGGTCACCGACAGGCATAAC | 122 |
| P16-R | CAGAAGTGAAGCCAAGGAGAAA |  |
| P27-F | TGGACCAAATGCCTGACTC | 144 |
| P27-R | GGGAACCGTCTGAAACATTTTC |  |
| P53-F | TCCGACTATACCACTATCCACTAC | 149 |
| P53-R | GCACAAACACGAACCTCAAAG |  |
| P21-F | TGTTCCACACAGGAGCAAAG | 175 |
| P21-R | AACACGCTCCCAGACGTAGT |  |
| Lamin B1-F | GAATTCTCAGGGAGAGGAGGTT | 149 |
| Lamin B1-R | TATTGGATGCTCTTGGGGTTC |  |
| mTOR-F | ACGCCTGCCATACTTGAGTC | 184 |
| mTOR-R | TCGTGTCCATCTTCTTGTCG |  |
| FOXO3-F | CGGCTCACTTTGTCCCAGAT | 163 |
| FOXO3-R | TCTTGCCAGTCCCTTCGTTC |  |
| Sirt1-F | AAGGAGCAGATTAGTAAGC | 118 |
| Sirt1-R | TAGAGGATAAGGCGTCAT |  |
| IL-1β-F | TGCAGGCTTCGAGATGAAC | 149 |
| IL-1β-R | GGGATTTTGTCGTTGCTTGTC |  |
| IL-6-F | TCCTACCCCAACTTCCAATGCTC | 79 |
| IL-6-R | TTGGATGGTCTTGGTCCTTAGCC |  |
| TNFα-F | CTTCTCATTCCTGCTCGTGG | 142 |
| TNFα-R | TGATCTGAGTGTGAGGGTCTG |  |
| IL-18-F | TGGAGACTTGGAATCAGACC | 398 |
| IL-18-R | GGCAAGCTAGAAAGTGTCCT |  |
