# Supplementary material for: Young cardiac telocyte-derived exosomes rejuvenate aging hearts in rats
Source: Front Cell Dev Biol. 2026 Jul 9;14:1824533. doi: 10.3389/fcell.2026.1824533 (PMC13391955; doi:10.3389/fcell.2026.1824533)
Supplement: Supplementary file 4 [file Table2.docx]

**Supplementary Table 2：Transcriptomic sequencing read counts and alignment analysis of cardiac tissue**

| Sample | Total clean reads | Total mapped reads | Mapping ratio |
| --- | --- | --- | --- |
| PBS_P653 | 50201544 | 48925174 | 97.46% |
| PBS_P654 | 45970862 | 44680335 | 97.19% |
| PBS_P676 | 51108410 | 49756154 | 97.35% |
| PBS_P869 | 49249098 | 47878722 | 97.22% |
| EXO_P657 | 48347692 | 47084633 | 97.39% |
| EXO_P667 | 47070278 | 45898385 | 97.51% |
| EXO_P863 | 47221556 | 46031093 | 97.48% |
| EXO_P870 | 47210076 | 46012359 | 97.46% |

|  |
| --- |

|  |
| --- |
|  |

|  |
| --- |
